# Exposure to Endocrine Disrupting Chemicals in Canada: Population-Based Estimates of Disease Burden and Economic Costs

**DOI:** 10.3390/toxics10030146

**Published:** 2022-03-19

**Authors:** Julia Malits, Mrudula Naidu, Leonardo Trasande

**Affiliations:** 1 Harvard Medical School, Boston, MA 02115, USA; 2 Department of Pediatrics, NYU School of Medicine, New York, NY 10016, USA; mrudula.naidu@nyulangone.org (M.N.); leonardo.trasande@nyulangone.org (L.T.); 3 Department of Environmental Medicine, NYU School of Medicine, New York, NY 10016, USA; 4 Department of Population Health, NYU School of Medicine, New York, NY 10016, USA; 5 NYU Wagner School of Public Service, New York, NY 10012, USA; 6 NYU School of Global Public Health, New York, NY 10003, USA

**Keywords:** endocrine-disrupting chemicals, Canada, disease burden, economic costs, GDP

## Abstract

Exposure to endocrine-disrupting chemicals (EDCs) contributes to substantial disease burden worldwide. We aim to quantify the disease burden and costs of EDC exposure in Canada and to compare these results with previously published findings in the European Union (EU) and United States (US). EDC biomonitoring data from the Canadian Health Measures Survey (2007–2011) was applied to 15 exposure–response relationships, and population and cost estimates were based on the 2010 general Canadian population. EDC exposure in Canada (CAD 24.6 billion) resulted in substantially lower costs than the US (USD 340 billion) and EU (USD 217 billion). Nonetheless, our findings suggest that EDC exposure contributes to substantial and costly disease burden in Canada, amounting to 1.25% of the annual Canadian gross domestic product. As in the US, exposure to polybrominated diphenyl ethers was the greatest contributor of costs (8.8 billion for 374,395 lost IQ points and 2.6 billion for 1610 cases of intellectual disability). In the EU, organophosphate pesticides were the largest contributor to costs (USD 121 billion). While the burden of EDC exposure is greater in the US and EU, there remains a similar need for stronger EDC regulatory action in Canada beyond the current framework of the Canadian Environmental Protection Act of 1999.

## 1. Introduction

In the past several decades, international efforts to improve our understanding of the adverse health outcomes of exposure to endocrine disrupting chemicals (EDCs) have markedly expanded [1]. A growing body of literature continues to document the varied and harmful effects that many widely used man-made chemicals have upon the endocrine system [2,3]. Even more so, low-income and marginalized communities are known to be disproportionately exposed to such chemicals [4,5].

In its first of two scientific statements, the Endocrine Society submitted that “from a physiological perspective, an endocrine-disrupting substance is a compound, either natural or synthetic, which, through environmental or inappropriate developmental exposures, alters the hormonal and homeostatic systems that enable the organism to communicate with and respond to its environment” [6]. EDCs encompass many substances, including polychlorinated biphenyls, polybrominated diphenyl ethers, dioxins, bisphenols, phthalates, among others. They are used for a range of purposes, such as industrial solvents and lubricants, plastics, plasticizers, pesticides and pharmaceutical agents. ^6^ Adverse health outcomes associated with exposure to EDCs include, but are not limited to, testicular and breast cancer, infertility, male and female reproductive dysfunction, birth defects, obesity, diabetes, cardiovascular and pulmonary disease, as well as neurobehavioral disorders [7]. Recent studies provide an overview of the mechanisms of action of EDCs, which include inhibitory or stimulatory binding to a hormone receptor, stimulation or inhibition of endogenous hormone production or hormone receptor expression or epigenetic effects, all of which may lead to a disruption in the endocrine system [3,8].

Previously published studies by our group have quantified the disease burden and economic costs associated with EDCs in the European Union (EU) and United States (US). The authors examined EDCs (polybrominated diphenyl ethers (PBDE), organophosphate pesticides (OP), dichlorodipheyldichloroethylene (DDE), bisphenol A (BPA), and phthalates) and associated health outcomes for which a steering committee of experts found robust toxicological and epidemiologic evidence [9]. Our group found that EDCs amounted to USD 217 billion (1.28% of gross domestic product, GDP) in the EU and USD 340 billion in the US (2.33% of GDP) [9,10]. In Canada, the epidemiologic literature evaluating the disease burden of environmental chemicals, including EDCs, is growing [11]. Eykelbosh and colleagues (2018) provide a systematic review of recent studies evaluating exposure to environmental chemicals in the general Canadian population using data from the Canadian Health Measures Survey (CHMS). For example, Do and colleagues (2017) applied the CHMS to demonstrate a positive association between bisphenol A (BPA) exposure and obesity in Canada, an association that has been widely corroborated by other studies [12,13]. However, there is limited evidence of the economic costs due to the EDC-attributable disease burden in Canada.

The principal regulatory framework for environmental substances, including EDCs, is the Canadian Environmental Protection Act (CEPA) of 1999, though there is no explicit mention of EDCs. To manage EDC exposure, both the US and Canada apply a risk-based approach. In contrast, the EU applies the precautionary principle in its approach to regulating EDCs and other environmental chemicals [4]. This analysis is the most comprehensive study to date documenting the disease burden and economic costs associated with exposure to multiple EDCs in the general Canadian population. The aim of this study is to quantify the disease burden and economic costs associated with exposure to EDCs in Canada, and to place our findings in the context of previously published studies in the US and EU.

## 2. Materials and Methods

### 2.1. Study Design

This study was designed in parallel to our previous publications investigating population-based estimates of EDC—attributable disease burden in the EU and US. We applied the ranges for probabilities of causation put forth by expert panels established by the Endocrine Society intended to assess the disease burden and economic costs attributable to exposure to EDCs in Europe [9]. The probabilities were determined based on available laboratory and epidemiological evidence, the strength of which was appraised using the Danish Environmental Protection Agency criteria and the GRADE Working Group criteria, respectively [14,15]. A scientific steering committee adapted the approach developed by the Intergovernmental Panel on Climate Change (IPCC, Geneva, Switzerland) to generate probabilities of causation based on the strength of the laboratory and epidemiological evidence [16].

The laboratory and toxicological evidence pertained to 15 exposure–response relationships between EDCs and various diseases. The EDCs evaluated were polybrominated diphenyl ethers (PBDE), organophosphate pesticides (OP), dichlorodipheyldichloroethylene (DDE), bisphenol A (BPA), phthalates (di-2-ethylhexylphthalate (DEHP), benzylphthalates, butylphthaltes) and combinations of these chemicals (see Supplementary Materials). The categories for health outcomes were neurodevelopmental dysfunction (loss of intelligence quotient points and resultant intellectual disability, attention deficit hyperactivity disorder, autism), metabolic disorders (adult and childhood obesity, adult diabetes), male reproductive disorders (cryptorchidism, testicular cancer, infertility, early cardiovascular mortality due to decreased testosterone levels) and female reproductive disorders (leiomyomas and endometriosis).

To estimate the cost of disease burden attributable to environmental exposures, we applied a model used by the Institute of Medicine [17], as we had in our EU and US studies. The model is as follows:

Attributable disease burden = increment in disease × attributable fraction × population size.

Attributable cost = increment in disease × attributable fraction × population size x cost per increment.

The attributable fraction (AF) of a risk factor is defined as the proportional decrease in the number of cases of morbidity or mortality resulting from a decrease in the risk factor [18]. The AF can be quantified by the equation:

$$\mathrm{Attributable}\;\mathrm{fraction}=\frac{{\mathrm{prevalence}}_{\mathrm{exposure}}\times (\mathrm{RR}-1)}{1+\left({\mathrm{prevalence}}_{\mathrm{exposure}}\times (\mathrm{RR}-1)\right)}$$

where relative risk (RR) is the relative risk of morbidity associated with a particular exposure.

### 2.2. Data Collection and Measurements

To create estimates comparable to those for our EU and US studies, we obtained nationally representative human biomonitoring data from the CHMS, which is jointly overseen by Statistics Canada, Health Canada and the Public Health Agency of Canada. CHMS has been continuously administered since 2007 in two-year cycles. Further information about CHMS has been documented extensively by Haines and colleagues (2017) [19]. When biomonitoring data for specific substances was not included in CHMS, we applied data from the National Health and Nutrition Examination Surveys (NHANES) to extrapolate the expected levels in the general Canadian population based on the appropriate ratios of chemicals. NHANES is a nationally representative, multicomponent survey of the non-institutionalized US population that is administered biennially by the National Centers for Health Statistics of the Centers for Disease Control and Prevention. Further, when specific percentiles were missing from the dataset, we interpolated the appropriate exposure level.

Data for PBDE, OP and DDE was extracted from the 2007–2009 survey cycles, and from the 2009–2011 survey cycles for BPA and phthalates. Values for all chemicals were separated into percentile ranges: 0–9th, 10th–24th, 25th–49th, 50th–75th, 75th–89th, and 90th–99th. The lowest quintile was assumed to have no exposure, and the remaining percentiles were assumed to have levels of exposure corresponding to their respective lowest extreme (i.e., 10th percentile of exposure for all individuals in the 10th–24th percentile grouping). Census data for the Canadian population, stratified by gender and age, was obtained from the Census Program, coordinated by Statistics Canada every five years [20]. We applied census data from 2010 with one exception (women married or living in common law in 2011 for phthalate-associated increases in assisted reproductive technology).

### 2.3. Exposure-Response Relationships

We considered four ERRs relating to neurobehavioral dysfunction (PBDE and intellectual quotient (IQ) point loss and intellectual disability (ID), OP and IQ point loss and ID, multiple exposures (OP and PBDE) and ADHD, phthalates and autism); five ERRs related to metabolic dysfunction (DDE and childhood obesity, DDE and adult diabetes, DEHP and adult obesity, DEHP and adult diabetes, BPA and childhood obesity); three ERRs related to male reproductive dysfunction (PBDE and testicular cancer, PBDE and cryptorchidism, phthalates and male infertility resulting in assisted reproductive technology (ART), phthalates and low testosterone resulting in increased early mortality); and two ERRs related to female reproductive dysfunction (DDE and leiomyomas, DEHP and endometriosis). Whenever possible, we applied exposure–response relationships identified by studies focused on EDC exposure levels of the general Canadian population in our base case estimate and sensitivity analysis. When Canadian studies were unavailable in the literature, we used the exposure–response relationships previously applied in the EU and US studies. Our methodological approach for each exposure–response relationship is discussed in detail in the Supplementary Materials.

### 2.4. Estimates of Economic Costs

We estimated total economic costs for each disease by applying a cost-of-illness approach for both direct costs and indirect costs [21]. Similar to our US study, we followed previously published guidelines by the Panel on Cost Effectiveness and Medicine [22]. We applied Canadian data sources and published cost estimates whenever possible for base case estimates, as well as low-end and high-end estimates, in our sensitivity analysis. When Canadian estimates were unavailable, we used data from NHANES and the cost estimates previously applied to our US study. Overall costs were generated by applying the disease incidence or prevalence and the size of the at-risk population. All economic costs were adjusted to the 2010 Canadian dollar year using the medical care consumer price index [23], and converted from the US to Canadian dollar using purchasing power parities (PPP) [24]. A detailed summary of the publications used for cost estimates may be found in the Supplementary Materials.

### 2.5. Statistical Analysis

We performed a descriptive analysis using Microsoft Excel and Stata 14.1. When biomonitoring data from NHANES was applied to extrapolate phthalate values in the Canadian general population, the appropriate environmental sample weights for subsamples were incorporated for the years 2009–2010.

## 3. Results

A summary of the disease burden and economic costs attributable to EDCs for each exposure–response relationship along with sensitivity analyses is presented in Table 1 and Table 2, respectively. Overall, our findings indicate that the disease burden arising from EDC exposure is substantially lower in the general Canadian population than in either the US or EU.

As in our US study, we found that the greatest burden of disease and economic costs associated with EDC exposure in Canada was neurobehavioral dysfunction resulting from PBDE exposure. Specifically, in utero PBDE exposure was associated with 374,395 lost IQ points and 1610 ID cases, and consequently, $8.8 billion CAD in lost IQ points and $2.6 billion CAD resulting from ID. The second largest contributor to EDC-attributable disease burden was phthalate exposure leading to endometriosis. Exposure to DEHP was associated with 10,151 cases of endometriosis, resulting in $5.7 billion CAD in direct and indirect costs. 

Of the $24.6 billion CAD in total costs attributable to EDC exposure in Canada, $11.5 billion resulted from exposure to PBDE, $8.4 billion from phthalates, $4.3 billion from organophosphate pesticides, $391 million from DDE, and $59 million from BPA. Of all adverse health outcomes attributable to EDC exposure, neurobehavioral dysfunction accounted for $15.9 billion, metabolic disorders (diabetes and obesity) in children and adults accounted for $1.2 billion, male reproductive disorders (testicular cancer, cryptorchidism and infertility leading to use of assisted reproductive technology) accounted for $30.1 million, early mortality associated with decreased testosterone levels accounted for $1.8 billion, and female reproductive disorders (endometriosis and uterine fibroids) accounted for $5.7 billion.

A comparison of our findings in the US, EU and Canada is presented in Table 3. The most notable trend in Table 3 is that the disease burden and associated costs of EDC exposure are substantially more extensive in the US and EU than in Canada for all exposure–response relationships considered in this study. In our initial study evaluating EDC-attributable disease burden in the EU, we found that exposure to organophosphate pesticides resulted in the greatest number of cases and associated costs. In the US, however, our analysis revealed that exposure to PBDE played the largest role in EDC-attributable disease burden and associated costs.

## 4. Discussion

In our analysis, the costs of exposure to EDCs among the general Canadian population amounted to $24.6 billion (CAD 2010). This amounts to 1.25% of the Canadian GDP in 2010 or $724 CAD per capita [26]. In contrast, EDC exposure was associated with $340 billion USD in the US (2.33% of US GDP) and $217 billion USD in the EU (1.28% of EU GDP). The main driver of costs associated with EDC exposure in Canada was PBDE-associated IQ loss and intellectual disability (ID), resulting in $11.5 billion CAD, or $276.5 USD per capita in Canada. This is three times lower than PBDE-associated IQ loss and ID in the US, which amounted to $266 billion USD or $860 USD per capita. PBDE-associated IQ loss and ID in the EU, with $12.6 billion USD in economic costs or $28.5 USD per capita, were much lower than in Canada.

Our findings must be understood within the current regulatory environment in Canada regarding EDCs. The differences likely relate to policy differences between North America and Europe, in which restrictions on PBDEs were greater in Europe, especially compared to the US where their use was essentially required to meet a California flammability standard which has since been revised [10]. As in the US, Canada approaches chemical regulation with a risk-based strategy under the Canadian Environmental Protection Act of 1999 [27]. Within CEPA 1999, there is no explicit regulation of EDCs; rather, a risk assessment similar to other synthetic compounds is performed. In comparison, the EU applies the precautionary principle, the more robust and prudent strategic approach [4].

We acknowledge several limitations in this study, some of which are similar to those outlined in our EU and US analyses. We closely followed the rigorous methodology of our prior studies to review and apply the toxicological and epidemiological evidence in this analysis [9,28]. However, we recognize that strong toxicologic and epidemiologic evidence supporting an association between EDC exposure and adverse health outcomes, as well as their underlying pathophysiologic mechanisms, far outweigh expert opinion. Nonetheless, the available literature to date is both robust [8] and speaks to the importance of urgently addressing the disease burden and costs associated with EDC exposure in Canada and globally. This analysis, moreover, excluded Monte Carlo simulations, as the central aim of our study was principally to compare the disease burden and associated costs across several countries. To account for uncertainty in our estimates, Canadian policymakers may multiply the aggregate costs by a factor of 0.8 for each exposure–response relationship.

Further, we recognize that our study focused on evaluating the effects of exposure to a multitude of chemicals individually, rather than the effects of combined exposure to EDCs. While this was not the aim of our study, we emphasize that it remains an important area of ongoing scientific inquiry [29]. Our study likely underestimates the true burden of disease and economic costs to society associated with EDC exposure for at least three reasons. First, our study assessed <5% of all EDCs for which there is adequately robust exposure, toxicological and epidemiological evidence to meet the criteria for inclusion in our analysis [30]. For similar reasons, we only assessed those EDC-associated health outcomes for which there is solid, convincing evidence for causation. There is likely a far larger constellation of diseases and economic costs associated with EDC exposure than is reflected in our study, which may very well underestimate the true economic costs and disease burden borne by the Canadian population [31]. Lastly, our economic estimates reflect both healthcare-associated and indirect (i.e., DALYs) costs that likely do not fully capture the economic toll borne by individuals suffering from the morbidity associated with EDC exposure, including intangible costs to quality of life.

Additionally, we based lifetime costs of chronic diseases on annual cost estimates in certain exposure–response relationships. While this approach is widely performed, we acknowledge that it is an imprecise estimate that would preferably be substituted with robust evidence of lifetime cost estimates if the data were available. Lastly, we recognize that the exposure data applied in this study dates to 2007–2011. While the most recent data would be more practical in guiding public health officials and policy makers in Canada, the timing of our data allows for a ready comparison of EDC exposure, disease burden and economic costs in Canada, the US and EU in the context of their regulatory environments based on our previously published studies. Lastly, it is likely that our analysis underestimates the disease burden and costs associated with more recent EDC exposure as the commercial applications of EDCs continue to rise globally [32].

The urgent public health threat of EDC exposure has been recognized by the Endocrine Society, the WHO and UNEP [7,33]. The gravity of this threat is underscored by evidence to suggest that EDCs have transgenerational effects on human health and well-being, and by extension, the economic health of society [34]. However, when considering alternatives to EDCs in commercial and industrial settings, it is critical to ensure that substitutions are validated to be safe and not simply a “regrettable substitution” [35].

To our knowledge, this study is the first to comprehensively examine multiple EDC exposures, disease burden and economic costs in Canada. Our findings underscore the urgent need to minimize EDC exposure among the general Canadian population to limit the substantial disease burden and economic costs to society, which amount to 1.25% the 2010 Canadian GDP.

We encourage more robust regulatory action that extends beyond CEPA 1999 to specifically and rigorously monitor and limit exposure to EDCs. In 2021, the Canadian federal government considered Bill C-28, which sought to modernize aspects of CEPA for the first time since its creation in 1999 [36]. In short, the bill aimed to modernize the Canadian government’s approach to promoting environmental health by recognizing every Canadian citizen’s right to a safe and healthy environment. The bill would have provided a regulatory framework for a more thorough reviewing of the toxicological and epidemiological evidence of various substances to act upon the available literature in a risk-based manner, and with a particular focus on identifying vulnerable populations. The bill further sought to amend the Food and Drugs Act to include a risk assessment of potentially toxic substances in food, drugs and personal care products—a critical regulatory step as these are substantial sources of human exposure to EDCs. While Bill C-28 stalled in the Fall 2021 parliamentary session, the need to modernize CEPA 1999 remains important. However, the bill did not make explicit mention of EDCs, an omission that we hope will be corrected in subsequent parliamentary sessions that reconsider Bill C-28 or introduce other bills to modernize CEPA 1999. If targeted regulatory steps are not taken, EDC exposure will continue to substantially contribute to disease burden and economic costs across the general Canadian population, especially among vulnerable communities.

## Figures and Tables

**Table 1:** Attributable burden of disease for 15 exposure–response relationships in Canada.

Exposure Response Relationship	Target Population	Base Case Estimate	Sensitivity Analyses
**Neurodevelopmental deficits**			
PBDE and IQ points loss and intellectual disability	All neonates	374,395 IQ points lost; 1610 ID cases	790,865–925,481 IQ points lost; 3674–4491 ID cases
OP and IQ points loss and intellectual disability	All neonates	152,922 IQ points lost; 377 ID cases	50,014–201,497 IQ points lost; 111–522 ID cases
Multiple exposures and ADHD	Children aged 12 years	180 cases (OPs)	329 cases (PBDE)
Phthalates and autism	Children aged 8 years	118 cases in males, 28 cases in females	47–236 cases in boys, 11–56 cases in girls
**Metabolic disorders**			
DDE and childhood obesity	Children aged 10 years	114 cases	318 cases
DDE and adult diabetes	Adults aged 40–59 years	3270 cases	36,209 cases
DEHP and adult obesity	Women aged 40–59 years	2093 cases	NA
DEHP and adult diabetes	Women aged 40–59 years	225 cases	NA
BPA and childhood obesity	Children aged 4 years	1023 cases	711 cases
**Male reproductive disorders**			
PBDE and testicular cancer	Men aged 20–79 years	316 cases	96–423 cases
PBDE and cryptorchidism	All male neonates	567 cases	NA
Phthalates and male infertility resulting in increased assisted reproductive technology	Men aged 20–39 years	1395 cases	NA
Phthalates and low testosterone resulting in increased early mortality	Men aged 60–79 years	3385 cases	NA
**Female reproductive disorders**			
DDE and fibroids	Women aged 15–54 years	2254 cases	NA
DEHP and endometriosis	Women aged 20–39 years	10,151 cases	NA

PBDE = polybrominated diphenyl ethers, OP = organophosphate pesticides, DDE = dichlorodiphenyldichloroethylene, DEHP = di-2-ethylhexylphthalate, IQ = intellectual quotient, NA = alternative inputs not available for sensitivity analyses, ADHD = attention deficit hyperactivity disorder.

**Table 2:** Cost estimates (2010 CAD) for disorders associated with exposure to EDCs in Canada.

Exposure Response Relationship	Base Case Estimate	Sensitivity Analysis: Low-End Estimate	Sensitivity Analysis: High-End Estimate or Alternative Scenario
**Neurodevelopmental deficits**			
PBDE and IQ points loss and intellectual disability	$8.8 billion (IQ); $2.6 billion (ID)	NA	$21.8 billion (IQ); $7.4 billion (ID)
OP and IQ points loss and intellectual disability	$3.6 billion (IQ); $619 million (ID)	$1.2 billion (IQ); $182 million (ID)	$4.7 billion (IQ); $858 million (ID)
Multiple exposures and ADHD	$34.8 million	$28.4 million	$75.4 million
Multiple exposures and autism	$188.2 million for boys, $44.7 million for girls	$75.3 million for boys, $17.9 million for girls	$376.5 million for boys, $89.5 million for girls
**Metabolic disorders**			
DDE and childhood obesity	$2.5 million	NA	$6.9 million
DDE and adult diabetes	$385.2 million	NA	$4.3 billion
DEHP and adult obesity	$684.8 million	NA	NA
DEHP and adult diabetes	$25.8 million	NA	NA
BPA and childhood obesity	$59 million	$41 million	NA
**Male reproductive disorders**			
PBDE and testicular cancer	$7.3 million	$2.2 million	$9.8 million
PBDE and cryptorchidism	$5.8 million	NA	NA
Phthalates and male infertility resulting in increased assisted reproductive technology	$17.0 million	NA	NA
Phthalates and low testosterone resulting in increased early mortality	$1.8 billion	NA	NA
**Female reproductive disorders**			
DDE and fibroids	$4.2 million	NA	NA
DEHP and endometriosis	$5.7 billion	NA	NA
**Total**	**$24.6 billion**	**NA**	**NA**

All cost estimates are reported in the 2010 Canadian dollar.

**Table 3:** Comparison of attributable disease burden and economic costs (base case estimates, 2010 USD) in the US, EU and Canada with 2010 population estimates.

	USA		EU		Canada	
Exposure-Response Relationship	Disease Burden	Economic Costs (USD)	Disease Burden	Economic Costs (USD)	Disease Burden	Economic Costs (USD)
**Neurodevelopmental deficits**						
PBDE and IQ points loss and intellectual disability	11 million IQ points lost; 43,000 ID cases	$266 billion	873,000 IQ points lost; 3290 ID cases	$12.6 billion	374,000 IQ points lost; 1610 ID cases	$7.2 billion (IQ); $2.2 billion (ID)
OP and IQ points loss and intellectual disability	1.8 million IQ points lost; 7500 ID cases	$44.7 billion	13 million IQ points lost; 59,300 ID cases	$194.0 billion	153,000 IQ points lost; 377 ID cases	$3.0 billion (IQ); $507 million (ID)
Multiple exposures and ADHD	4400 cases	$698.0 million	19,400–31,200 cases	$2.3 billion	180 cases	$28.5 million
Multiple exposures and autism	787 cases in boys, 754 cases in girls	$1.0 billion in boys, $984.0 million in girls	316 cases	$265.1 million	118 cases in boys, 28 cases in girls	$154.2 million for boys, $36.6 million for girls
**Metabolic disorders**						
DDE and childhood obesity	857 cases	$29.6 million	1555 cases	$32.7 million	114 cases	$2.1 million
DDE and adult diabetes	24,900 cases	$1.8 billion	28,200 cases	$1.1 billion	3270 cases	$315.4 million
DEHP and adult obesity	5900 cases	$1.7 billion	53,900 cases	$20.8 billion	2093 cases	$560.9 million
DEHP and adult diabetes	1300 cases	$91.4 million	20,500 cases	$807.2 million	225 cases	$21.2 million
BPA and childhood obesity	33,000 cases	$2.4 billion	42,400 cases	$2.0 billion	1023 cases	$48.3 million
**Male reproductive disorders**						
PBDE and testicular cancer	3600 cases	$81.5 million	6830 cases	$1.1 billion	316 cases	$6.0 million
PBDE and cryptorchidism	4300 cases	$35.7 million	4615 cases	$172.6 million	567 cases	$ 4.8 million
Phthalates and male infertility resulting in increased assisted reproductive technology	240,100 cases	$2.5 billion	618,000 cases	$6.3 billion	1395 cases	$13.9 million
Phthalates and low testosterone resulting in increased early mortality	10,700 attributable deaths	$8.8 billion	24,800 attributable deaths	$10.6 billion	3385 attributable deaths	$1.5 billion
**Female reproductive disorders**						
DDE and fibroids	37,000 cases	$259.0 million	56,700 cases	$216.8 million	2254 cases	$3.5 million
DEHP and endometriosis	86,000 cases	$47.0 billion	145,000 cases	$1.7 billion	10,151 cases	$4.6 billion

Exchange rate €1 = $1.33 USD; $1 USD = $1.221 CAD via PPP. Data for population estimates obtained from World Bank [25].

## Data Availability

Data regarding EDC exposure was obtained from the publicly available Canadian Health Measures Survey.

## Supplementary Materials

The supporting supplementary data can be downloaded at: www.mdpi.com/article/10.3390/toxics10030146/s1. References [37,38,39,40,41,42,43,44,45,46,47,48,49,50,51,52,53,54,55,56,57,58,59,60,61,62,63,64,65,66,67,68,69,70,71,72,73,74,75,76,77,78,79,80,81,82,83,84,85,86,87,88,89,90,91,92,93,94,95,96,97,98,99,100,101,102,103] is in the Supplementary Materials.
