# Supplementary material for: Exposure to Endocrine Disrupting Chemicals in Canada: Population-Based Estimates of Disease Burden and Economic Costs
Source: Toxics. 2022 Mar 19;10(3):146. doi: 10.3390/toxics10030146 (PMC8948756; doi:10.3390/toxics10030146)
Supplement: Supplementary file 1 [file toxics-10-00146-s001.zip › toxics-1591549-supplementary.pdf]

toxics

MDPI

Supplementary Materials: Exposure to Endocrine Disrupting Chemicals in Canada: Population-Based Estimates of Disease Burden and Economic Costs

Julia Malits, Mrudula Naidu and Leonardo Trasande

Methods

Overall Methodological Approach

As we had done for the previously published European and US analyses, we applied suggested ranges for the probability of causation based upon the strength of the toxicologic and epidemiologic evidence that were identified by a steering committee of scientists convened for the European analysis. The results of the steering committee can be found in the Appendix of our previously published European analysis [1].

Measurements

A summary of the endocrine-disrupting chemicals (EDCs) examined in this analysis can be found in Table 1. Measurements for EDCs were obtained from the 2009–2011 survey cycles of the Canadian Health Measures Survey (CHMS) in order to align with our previously published US and EU studies examining 2010 data. However, data from CHMS for polybrominated diphenyl ethers (PBDE), organophosphate pesticides (OP), and dichlorodiphenyldichloroethylene (DDE) were unavailable in the 2009–2011 survey cycles. As a result, their corresponding data was extracted from the 2007–2009 survey cycles.

Details of the underlying methodology for collecting and analyzing environmental chemicals under consideration in CHMS have been previously outlined [2]. When specific measurements were unavailable in the CHMS dataset, we applied the corresponding exposure values from NHANES to interpolate CHMS values. An elaboration of our interpolation method can be found below in the specific exposure–response relationships in which this approach was required.

1. Methods for Neurodevelopmental Deficit and Disability Burden

1.1. Modeling PBDE-Attributable IQ Deficits and Intellectual Disability

1.1.1. Exposure–Response Relationship

As in our US study, we applied a longitudinal birth cohort study documenting the association between a ten-fold increase in the in utero PBDE exposure and IQ loss (4.5 IQ points lost per log unit of PBDE) for our base case estimate [3] as well as two other cohort studies (4.7; 5.5 IQ points) for the sensitivity analysis. Likewise, we applied the reference level derived by Chen and colleagues for our base case estimate (6.4 ng/g lipid, corresponding to the 10th percentile). For the sensitivity analysis, we applied a reference level of 2.82 ng/g lipid as we had previously done in our US and EU studies.

We applied lipid-adjusted plasma levels of PBDE-47, which were subdivided into quintiles, for women 20–39 years of age in the 2007-2009 survey cycle of CHMS. This distribution of PBDE-47 levels was assumed to be identical to that of pregnant women in Canada in 2010. Census data for the number of births in Canada in 2010 was obtained and stratified by percentile ranges [4]. To calculate the IQ loss in each exposure interval, the exposure-associated IQ point loss was multiplied by the number of births in each percentile. The total IQ point loss attributable to in utero PBDE exposure was estimated as the sum of IQ point loss across all percentiles.

To estimate the number of intellectual disability (ID, defined as IQ < 70) cases attributable to in utero PBDE-47 exposure, we used the NORMDIST function in Microsoft Excel for the base case estimate and sensitivity analysis. Assuming a normal distribution with a mean IQ of 100 and SD of 15, we calculated the increment in additional ID cases due to IQ decrements associated with PBDE-47 exposure in each percentile. The change in increment of ID, as a percentage, was then multiplied by the population estimate across all percentiles. The sum of these values was estimated to be the total number of ID cases attributable to PBDE exposure.

1.1.2. Economic Evaluation

We applied cost estimates previously used in our US study. The cost for each IQ point loss was valued at $19,269 (2010 USD) [5] and the annual cost of ID was valued at $870,000 (2000 USD) [6]. Final cost estimates were corrected to the 2010 Canadian dollar using the US Consumer Price Index and the Canadian purchasing power parity (PPP) [7].

1.2. Modeling OP-Associated IQ Loss and Intellectual Disability

1.2.1. Exposure–Response Relationship

Our methodological approach closely reflects our approach for PBDE-attributable IQ deficits and intellectual disability. As in our US study, we applied a study by Bellinger and colleagues for the base case estimate (4.25 IQ point loss per 10-fold increase in prenatal OP exposure) with a corresponding reference level of 65 nmol/L [8]. For the sensitivity analysis, we applied a study by Engel and colleagues for the low-end estimate (1.39 IQ point loss per 10-fold increase in prenatal OP exposure) and Bouchard and colleagues for the high-end estimate (5.6 IQ point loss per 10-fold increase in prenatal OP exposure) [9] [10]. We similarly applied a reference level of 65 nmol/L in our sensitivity analyses.

For the total dialkyl phosphate level, representing OPs, we obtained data for percentile ranges of DMTP, DMP, and DEP in women 20–39 years of age in the 2007–2009 CHMS survey cycle. We assumed that this distribution of OPs was identical to that of pregnant Canadian women in 2010. Census data for the number of births in Canada in 2010 was obtained and stratified by percentile ranges [4]. To calculate the IQ loss in each exposure interval, the exposure-associated IQ point loss was multiplied by the number of births in each percentile. The total IQ point loss attributable to in utero OP exposure was estimated as the sum of IQ point loss across all percentiles.

To estimate the number of intellectual disability (ID, defined as IQ < 70) cases attributable to prenatal OP exposure, we used the NORMDIST function in Microsoft Excel for the base case estimate and sensitivity analysis. Assuming a normal distribution with a mean IQ of 100 and SD of 15, we calculated the increment in additional ID cases due to IQ decrements associated with OP exposure in each percentile. The change in increment of ID, as a percentage, was then multiplied by the population estimate across all percentiles; the sum of these values was estimated to be the total number of ID cases attributable to OP exposure.

1.2.2. Economic Evaluation

We applied cost estimates previously used in our US study. The cost for each IQ point loss was valued at $19,269 (2010 USD) [5] and the annual cost of ID was valued at $870,000 (2000 USD) [6]. Final cost estimates were corrected to the 2010 Canadian dollar using the US Consumer Price Index [11] and the Canadian PPP [7].

1.3. Modeling Autism Attributable to Multiple Exposures

1.3.1. Exposure–Response Relationship

To quantify the number of cases of autism spectrum disorder (ASD) attributable to EDC exposure, we applied a study of prenatal phthalate exposure previously used in our US analysis [12]. The expert panel had previously extrapolated from the study by

Miodovnik and colleagues to identify a single EDC attributable fraction (AF). The base case AF was estimated to be 5% while AFs of 2% and 10% were employed in the sensitivity analyses.

To measure EDC exposure, we applied the following low molecular weight (LMW) phthalates from women 20–39 years of age in the 2009-2011 CHMS survey cycle: MEP, MIBP and MNBP. MEP was unavailable in CHMS, and as such, we interpolated its value in Canadian women by applying the corresponding NHANES values. Specifically, we derived the ratio of MEP to MIBP values across each percentile in NHANES and multiplied this ratio by the MIBP percentile values in CHMS. The LMW phthalate levels in this demographic were assumed to be identical to those in pregnant women in 2010. The 10th percentile of exposure was then applied as the reference level.

To estimate the number of ASD cases in Canada attributable to LMW phthalate exposure, we obtained the prevalence of ASD among male (23.9 per 1000) and female (6 per 1000) Canadian children [13]. We then multiplied the respective prevalence of ASD by the number of 8-year-old males and females, which was acquired from the 2010 Canadian census [4]. Finally, we multiplied each AF by the number of Canadian children with autism while accounting for diagnostic overlap between autism and ADHD.

1.3.2. Economic Evaluation

As in our US study, we applied a lifetime cost of $1.4 million (USD 2012) toward supporting an individual with an ASD without ID [14]. The cost estimates, reported separately for boys and girls, were corrected to the 2010 Canadian dollar using the US Consumer Price Index [11] and the Canadian PPP [7].

1.4. Modeling ADHD Attributable to Multiple Exposures

1.4.1. Exposure–Response Relationship

For the exposure–response relationships, we applied two studies previously used in our US study and reviewed by the neurodevelopment expert panel (Bouchard et al., 2011, Gascon et al., 2011). We focused on two distinct EDCs—PBDE and OP—given that prenatal exposure to a multitude of EDCs likely contributes to the development of ADHD in childhood. OPs were applied in the main analysis and PBDE-47 was applied in the sensitivity analysis, in line with our prior EU and US analyses. We obtained urinary levels of OPs and lipid-adjusted plasma levels of PBDE-47 from Canadian women 20–39 years of age in the 2007–2009 CHMS survey cycle; we assumed that this distribution was identical to those of pregnant women in Canada in 2010.

In the main analysis of OPs, we first calculated the ratio of total OPs in each percentile to the reference level—65 nmol/L—used to estimate IQ loss. We then calculated the log unit of this ratio, which was subsequently exponentiated by the OR (1.35) identified by Bouchard and colleagues [10]. To arrive at the AF, we input this OR into the Levin equation and aggregated the AF corresponding to each percentile into the final AF [15]. In the sensitivity analysis of PBDE-47, we applied an OR of 1.80 [16] along with an exposure prevalence of 10% and a threshold of 24.4. In accordance with our OP estimates, we then applied the OR to the Levin equation and aggregated the AF corresponding to each percentile into the final AF [15].

Finally, we calculated the total number of childhood ADHD cases in Canada attributable to EDC exposure in both the main and sensitivity analysis. To estimate the number of children in Canada with ADHD, we first obtained the total number of 12-year-old children in Canada from the 2010 Canadian census [4]. A subsequent review of the literature did not yield an ADHD prevalence among Canadian children. As a result, we extrapolated the prevalence of ADHD among Canadian children from a study conducted by Vasiliadis and colleagues, in which the reported ADHD prevalence was stratified by sex and geographic province (Manitoba, Nova Scotia, Ontario and Quebec) and standardized by age [17].

We estimated the ADHD prevalence among Canadian children by calculating the weighted average of each ADHD prevalence, stratified by province and sex, with inputs of the total number of male and female children in each of the four Canadian provinces obtained from the 2011 Canadian census [4], as province-specific census data was unavailable for the year 2010. The final ADHD prevalence was then multiplied by the number of 12-year-old children in Canada. To estimate the ADHD burden attributable to each EDC exposure, we then multiplied the final AF by the number of children in Canada with ADHD while accounting for the diagnostic overlap between autism and ADHD [18].

1.4.2. Economic Evaluation

As in our US study, we employed previously published estimates of the annual cost of ADHD per individual, which ranged between $12,005 and $17,458 (2005 USD) [19]. We applied the mean of the two values as the base case estimate with sensitivity analyses included as well. Additionally, we aggregated annual cost estimates across 10 years with 3% discounting and corrected the cost estimates to the 2010 Canadian dollar using the US Consumer Price Index [11] and Canadian PPP [7].

2. Methods for Metabolic Disorders

2.1. Modeling DDE-Attributable Adult Diabetes

2.1.1. Exposure–response relationship

We obtained DDE levels, stratified into quintiles, in Canadian adults 40–59 years from the 2007–2009 CHMS survey cycle, and assumed that this distribution was identical to that in Canadian adults in 2010. For the base case estimate, we applied a study that identified a significantly increased OR (1.25; 95% CI: 0.94, 1.66) for diabetes in the setting of elevated DDE levels in the highest quartile [20]. For the sensitivity analysis, we applied the findings of a different longitudinal study and assigned the annual increments in newly incident cases reported for a specific range of exposure to the corresponding percentile in either of the two respective higher ranges [21]. In both the base case estimate and sensitivity analysis, we applied the age-standardized incidence of diabetes in Canada, 5.6 per 1,000 in 2008–2009, to obtain the increment in diabetes cases attributable to DDE exposure [22]. The increment was then applied to the 75th and 90th percentiles of DDE exposure levels and the population estimate of adult men and women 40–59 years of age in Canada in 2010 [4]. However, given the likelihood that some individuals in the target population already had diabetes, we subtracted the Canadian diabetes prevalence (5.6% in 2008–2009) from our estimate to isolate the disease burden and associated costs attributable only to DDE exposure [22].

2.1.2. Economic Evaluation

Per capita, direct cost estimates of diabetes were estimated as the average of two costs identified in a Canadian study, which reported an annual cost of $9,731 for men and $10,315 for women (CAD 2012) [23]. As in our US study, annual cost estimates were aggregated across 15 years with 3% discounting to account for the costs accrued over multiple years for those diagnosed with diabetes. Final cost estimates were corrected to the 2010 CAD using the Canadian Consumer Price Index [24].

2.2. Modeling DDE-Attributable Childhood Overweight/Obesity

2.2.1. Exposure–Response Relationship

As in our US study, we applied the exposure–response relationship identified by Iszatt and colleagues, who identified a 0.12 increment in the weight-for-age Z-score across the interquartile range [25]. We applied lipid-adjusted DDE levels for Canadian women 20–39 years of age from the 2007–2009 CHMS survey cycle, which was assumed to be identical to the distribution of DDE levels in pregnant Canadian women in 2010. The

NORMDIST function in Excel was used to convert the 0.12 change in the weight-for-age Z-score into a change in the proportion of infants with rapid early weight gain for each percentile. To do so, we assumed a threshold value at the 10th percentile, a normal distribution of Z-scores with a mean of 0 and SD of 1 and applied a cutoff value for rapid growth in infancy of 0.67 SD [26]. We then calculated the weighted sum of these values across each percentile of exposure. To determine the AF for exposed and unexposed scenarios, we then used the Levin formula [16] and a meta-analysis by Ong and colleagues, who identified that rapid weight gain in infancy was associated with a significantly increased OR (1.84) of overweight at age 10 [27]. The difference between the exposed and unexposed AF represented the increment in AF attributable to prenatal DDE exposure.

Next, we derived the distribution of overweight 10-year-old Canadian children, stratified into quintiles, using a prevalence of 19.5% for overweight status among 6-17-year-old Canadian children in 2007–2009 [28] and the number of 10-year-old Canadian children in 2010 [4]. The increment in AF was then multiplied by the number of overweight 10-year-old children in each percentile to determine the number of overweight cases in 10-year-old children attributable to prenatal DDE exposure.

For the sensitivity analysis, we applied a RR of 1.13 obtained from a linear relationship between rapid infant weight gain per log unit of maternal serum DDE level, which was identified in a prospective birth cohort study [29]. Our calculations were otherwise identical to the main analysis.

2.2.2. Economic Evaluation

We applied a meta-analysis that estimated $19,200 (2012 USD) as the lifetime social costs of obesity at age 10 [30]. This cost estimate accounts for childhood medical expenses [31,32] as well as adult medical expenses [33] and lost disability-adjusted life years (DALYs) associated with obesity in adulthood [34]. The final cost estimate was corrected to the 2010 Canadian dollar using the general US Consumer Price Index [11] and the Canadian PPP [7].

2.3. Modeling Phthalate-Attributable Adult Overweight/Obesity

2.3.1. Exposure–Response Relationship

As in our US study, we applied a longitudinal study that evaluated the association between exposure to phthalates and weight gain and obesity in females [35] to estimate annual weight gain attributable to phthalates. We obtained the distribution of percentiles of phthalate metabolites for DEHP (MEHHP, MEOHP, MEHP) as well as MBZP, MIBP, MNBP for Canadian women from 40 to 59 years of age from the 2009–2011 CHMS survey cycle. However, CHMS did not include data regarding levels of MECPP, a metabolite in the calculation of DEHP. To derive total DEHP levels in this Canadian population estimate, we obtained MECPP, MEOHP, MEHP and MEHHP levels for women from 40 to 59 years of age from NHANES in 2009–2010. We then multiplied the US MECPP value by the ratio of the sum of the three other phthalates in Canada and the US across each percentile in order to arrive at an interpolated MECPP value in Canadian women. This value was then added together with MEOHP, MEHHP and MEHP to estimate the total DEHP level.

Given that the analysis by Song and colleagues (2014) stratified its population by different percentiles from CHMS/NHANES, we used linear interpolations of the incremental weight gain from the 90th percentile and assigned an exposure value identical to the median of each percentile. We defined the midpoint of the highest percentile showing an insignificant association ($p > 0.05$) as the baseline level (OR = 1) below, which no effect was observed. To estimate the weight gain for each midpoint value, a linear fit of weight gain versus phthalate exposure for the percentiles above the baseline level was calculated for each exposure group.

We expressed weight gain attributable to phthalate exposure as a change in BMI. To express weight gain attributable to phthalate exposure as a function of the change in the mean BMI, we applied the mean BMI and SD for Canadian women from 40 to 59 years of age, which was obtained from a retrospective cohort study [36], and the mean height for Canadian women from 18 to 79 years of age [37]. The mean BMI was incorporated into the NORMDIST function in Excel to first obtain the pre-shift obesity (defined as BMI > 30 kg/m^2) prevalence. Similarly, the new mean BMI was incorporated into the NORMDIST function to obtain the obesity prevalence in the exposure scenario. We calculated the corresponding incremental change in obesity attributable to phthalate exposure by taking the difference between the pre-shift and post-shift obesity prevalence. To quantify the number of obesity cases attributable to phthalate exposure, this increment was multiplied by the pertinent population estimate. Census data for Canadian women from 40 to 59 years of age in 2010 was used for the population estimate [4].

2.3.2 Economic Evaluation

Direct cost estimates were obtained from a Canadian study that identified average physician costs of $105 annually for obese females (2003 CAD) [38]. Indirect cost estimates were unavailable in the Canadian literature, and as such, we applied estimated DALY losses due to overweight/obesity previously utilized in our US study [39]. DALY losses were multiplied by $50,000 USD, and ten years of discounting was applied for each DALY, as previously done in our US study. The total cost of overweight/obesity in Canadian women was estimated as the sum of direct and indirect cost expenditures. Final cost estimates were corrected to the 2010 Canadian dollar using the Canadian general Consumer Price Index [24] and PPP [7].

2.4. Modeling Phthalate-Attributable Adult Diabetes

2.4.1. Exposure–Response Relationship

We applied urinary levels of phthalate metabolites (MEHHP, MEHP, MEOHP) that together constitute DEHP for women 40–59 years of age in the 2009-2011 survey cycle of CHMS. However, CHMS did not include data regarding levels of MECPP, an additional metabolite in the calculation of DEHP. To interpolate a MECPP value in the corresponding Canadian population estimate, we followed an identical approach to that outlined for phthalate-attributable adult overweight/obesity above.

We applied an OR of the association between phthalate exposure and adult diabetes identified in the Nurses' Health Study II and previously used in our US study [40]. The median urinary phthalate concentration in the first quartile of the NHS II was applied as the reference level, which was exceeded only by the 90th percentile of exposure in our study. As in our earlier phthalate-attributable adult obesity analysis, we used linear interpolations of the incremental weight gain from the 90th percentile and assigned an exposure value identical to the median of each percentile. We defined the midpoint of the highest percentile showing an insignificant association ($p > 0.05$) as the baseline level (OR = 1) below, which no effect was observed. To estimate the weight gain for each midpoint value, a linear fit of weight gain versus phthalate exposure for the percentiles above the baseline level was calculated for each exposure group.

To calculate the number of diabetes cases attributable to phthalate exposure, we first obtained age-standardized incidence (5.6 per 1000) and prevalence (5.6%) of diabetes in Canada in 2008–2009 [22]. The appropriate OR was then multiplied by the annual incidence of diabetes to calculate the incident rate attributable to phthalate exposure. The baseline prevalence of diabetes was then subtracted from the phthalate-attributable incident rate of diabetes to arrive at an incremental incident rate attributable to phthalates. Finally, the incremental rate of diabetes was multiplied by the corresponding target population [4].

2.4.2. Economic Evaluation

Per capita, direct cost estimates of diabetes were obtained from a Canadian study, which reported an annual cost of $9,731 (CAD 2012) for women [23]. As in our US study, annual cost estimates were aggregated across 15 years with 3% discounting to account for the costs accrued over multiple years for those diagnosed with diabetes. The final cost estimate was corrected to the 2010 CAD using the Canadian Consumer Price Index [24].

2.5 Modeling BPA-Attributable Childhood Obesity

2.5.1. Exposure–Response Relationship

The typical target population of childbearing women applied in this study is females 20–39 years of age, but BPA levels in this demographic were unavailable in CHMS. Thus, we obtained the distribution of percentiles of urinary creatinine-corrected BPA levels among females 6–79 years of age in the 2009-2011 CHMS survey cycle, which was assumed to be identical to the BPA distribution among pregnant women in Canada in 2010.

As in our US study, we applied a study that identified an association between log-transformed prenatal urinary BPA levels and an OR (0.28) of linear increments in the BMI Z-score at age 4 [41] for the main analysis. As with our US study, the reference level was set at the 25th percentile. The incremental increase in BMI Z-scores was calculated for each percentile of exposure. To estimate the number of childhood obesity cases attributable to BPA exposure, we then modeled across the percentile ranges for the population of 4-year-old children in Canada in 2010 [4]. To estimate the number of childhood obesity cases that extend into adulthood, we applied a 50% persistence rate based on a previous publication [42].

In the sensitivity analysis, we applied a study by Vafeaidi and colleagues that identified an association between log-transformed BPA levels at age 4 and an increment (0.2) in the BMI Z-score [43]. The sensitivity analysis otherwise followed the same procedure described above in the main analysis.

2.5.2 Economic Evaluation

To estimate lifetime costs of childhood obesity at age 4, we first applied two Canadian studies: we obtained an estimate of the lifetime (from birth to 14 years old) physician costs of childhood obesity [44] and an estimate of the direct cost of obesity in adulthood [45]. For the lifetime physician costs of childhood obesity, we applied the cost difference between normal weight and obese children ($379 in CAD 2010) and applied 10 years of discounting at 3% [44]. For the direct cost of obesity in adulthood, we applied the cost difference between normal weight and obese adults, which Tarride et al. quantifies at $176.1 (2011 CAD) [45]. We applied 35 years of discounting at 3% to the direct costs of obesity beginning at age 4 and extending into adulthood.

Additionally, we included the indirect cost estimate of obesity in adulthood using lost DALYs (valued at $50,000 in 2005 USD) [34]. Final cost estimates with inputs from the primary Canadian studies were updated to the 2010 Canadian dollar using either the Canadian CPI [24] and the indirect costs were corrected using the Canadian currency exchange rate [7].

3. Methods For Reproductive Disorders

3.1. Methods for Male Reproductive Disorders

3.1.1. Modeling PBDE-Attributable Testicular Cancer

Exposure-Response Relationship

We applied a case-control study, previously applied in our US study, that identified a significantly increased OR (2.5, 95% CI:1.02–6.0) of testicular cancer in the setting of elevated maternal serum levels of the sum of three PBDEs (PBDE-47, PBDE-99, PBDE-153).

Specifically, this association was found at exposure levels of the median value of 3.66 ng/g lipid in the control group [46]. Indeed, the exposure prevalence above 3.66 ng/g lipid was found to be >99.5%. As such, we applied lipid-adjusted plasma levels of PBDE-47, which were subdivided into quintiles, for men 20–79 years of age in the 2007–2009 survey cycle of CHMS. The distribution of PBDE-47 levels in 20–79-year-old men in the 2007–2009 survey cycle in CHMS was assumed to be identical to that in Canadian men with testicular cancer in 2010. Census data for the population of Canadian men 20–79 years of age in 2010 was applied [4].

We applied an exposure prevalence of 50% for the base case estimate as well as 10% for low-end estimates and 90% for high-end estimates in the sensitivity analysis. To quantify the fraction of testicular cancer cases attributable to PBDE exposure, each exposure prevalence was then input into the Levin equation [16] along with the OR. Finally, the AFs for the base case estimate and sensitivity analysis were multiplied by the incidence of testicular cancer in Canada [47] and the relevant population estimates for 2010 in order to determine the number of testicular cancer cases attributable to PBDE exposure.

Economic Evaluation

Cost estimates were obtained from a Canadian study that identified 5-year direct costs of $22,919 (2009 CAD), discounted at 5%, for testicular cancer [48]. We updated the cost estimate to the 2010 Canadian dollar using the Canadian general Consumer Price Index [24].

3.1.2. Modeling PBDE-Attributable Cryptorchidism

Exposure–Response Relationship

We applied a case-control study, previously applied in our EU and US studies, that identified a significantly increased OR (5.6) of cryptorchidism in the setting of elevated PBDE in breast milk, a proxy for PBDE exposure during infancy [49]. We obtained lipid-adjusted plasma levels of PBDE-47, subdivided into quintiles, in women from 20 to 39 years of age in 2007–2009 from CHMS, assuming that this distribution of PBDE-47 levels was identical to that in pregnant women in 2010. To obtain PBDE levels in breast milk, a previously identified PBDE serum/milk ratio of 0.8 was applied across percentiles (assuming that serum levels were identical to plasma levels given that serum values were unavailable in CHMS) [50]. We then exponentiated the OR across percentiles of estimated PBDE levels in breast milk, applying a reference level at the 50th percentile of exposure as we did in our US study. To determine the incidence of PBDE-attributable cryptorchidism, the OR values for the 75th and 90th percentiles were multiplied by the incidence of congenital cryptorchidism (assumed to be 1% as in both the EU and US analyses) and the number of Canadian births (males) in 2010 [4].

Economic Evaluation

Cost estimates were obtained from an economic analysis evaluating the cost of infant versus post-pubertal orchiopexy in the US, which was likewise applied in our US study [51]. Cost estimates were corrected to the 2010 Canadian dollar using the US Consumer Price Index [11] and the Canadian PPP [7].

3.1.3. Modeling Phthalate-Associated Reductions in Testosterone and Associated All-Cause Mortality.

Exposure–Response Relationship

We obtained the distribution of percentiles of urinary DEHP and MnBP levels among males 40–59 years of age in the 2009–2011 CHMS survey cycle. However, CHMS did not include data regarding levels of MECPP, a metabolic component of DEHP. In order to derive total DEHP levels in this Canadian population estimate, we obtained MECPP,

MEOHP, MEHP and MEHHP levels for men of the same age range from NHANES in 2009–2010. We then multiplied the US MECPP value by the ratio of the sum of the three remaining phthalates in the Canadian and US datasets across each percentile, and thus, derived an interpolated MECPP value in Canadian men. This value was then added together with MEOHP, MEHHP and MEHP to estimate the total DEHP level among Canadian men.

For the exposure–response relationship, we applied the findings of a cross-sectional study, previously used in our US study, that estimated decrements in serum testosterone levels associated with urinary DEHP metabolites and MnBP levels among men aged 40-60 years old [52]. The authors identified a 12.9% and 7.84% decrement in serum testosterone levels per unit increase in MnBP and DEHP, respectively, based on the interquartile range of exposure in the US sample. These exposure–response relationships were then applied across the distribution of percentiles of exposure to MnBP and DEHP among Canadian men. The 10th percentile of exposure was applied as the reference value for both DEHP and MBP. For the remaining percentiles, we estimated decrements in serum testosterone levels associated with either MnBP or DEHP exposure, which were then aggregated to arrive at testosterone decrements attributable to total phthalate exposure.

To derive the number of attributable all-cause deaths, we applied a meta-analysis of eleven longitudinal studies that were previously applied in our US study [53]. The authors estimated a relative risk of 1.35 (95% CI:1.13–1.62) for all-cause mortality associated with decrements in testosterone levels, which were based on the difference between the highest and lowest tertiles of exposure. As testosterone levels among Canadian men were unavailable from a nationally representative source, we relied on the mean serum testosterone levels among men 40–64 years of age available in NHANES and previously used in our US study. We derived an increment in testosterone of 7.72 nmol/L between the highest and lowest tertiles, which was then applied to the relative risk identified by Meeker and Ferguson (2014) in order to estimate the increment in all-cause mortality per unit (nmol/L) decreases in serum testosterone. To estimate the relative risk of all-cause mortality due to phthalate-attributable decreases in testosterone, the increment was then applied across the distribution of percentiles of DEHP and MnBP exposure. We then applied the baseline age-standardized all-cause mortality rate (853.7 per 100,000) among Canadian men in 2010 [54] to the relative risk of all-cause mortality due to reduced testosterone to estimate the phthalate-exposed mortality rate. The baseline mortality rate was then subtracted to yield the increment in the all-cause mortality rate attributable to phthalate exposure. To estimate the number of annual deaths attributable to phthalate-associated reductions in testosterone, we then applied this increment to the population of Canadian men between the ages of 55 and 64 years old in 2010 [4].

Economic Evaluation

As in our US study, we applied the previously published lifetime loss in economic productivity due to death among 55–59 ($526,356 in 2009 USD) and 60–64-year-old ($316,498 in 2009 USD) individuals [55]. Final cost estimates were corrected to the 2010 CAD using the US CPI [11] and the Canadian PPP [7].

3.1.4. Phthalate-Associated Increases in Assisted Reproductive Technology

Exposure–Response Relationship

We obtained the distribution of percentiles of urinary MBZP and MnBP levels among Canadian men between 20–39 years of age from the 2009–2011 CHMS survey cycle and assumed that these values were identical to those of male partners of child-bearing age women who were either married or living in common law. The lowest percentile of exposure (0–9th) was assumed to have no exposure, while the remaining percentiles were assumed to have an exposure corresponding to the lower bound of the range (i.e. we applied the 10th percentile in the 10th–24th percentile group).

As in our US study, we applied the results of the LIFE Study, a longitudinal study that identified an association between urinary phthalates in males and time-to-pregnancy (TTP). This association enabled us to quantify the number of Canadian couples who sought assisted reproductive technology (ART) due to decreased male fertility, reflected by a TTP > 12 months [56]. We then applied the results from (1) the Canadian ART Register [57], which identified that 13,713 individual women received ART in 2010; and, (2) a Canadian cross-sectional study that identified 365,100 Canadian couples who "reported no pregnancy, did not use any form of birth control, reported having sexual intercourse during the previous 12 months and had tried at some point to become pregnant with their current partner" [58]. Taken together, we estimated that 3.8% of infertile couples in Canada sought ART services.

Next, we applied the fecundity ORs identified in the LIFE Study for MBZP (0.82) and MnBP (0.77) across the exposure distribution by exponentiating the ratio of each phthalate percentile to the reference level of 0.2 ng/mL, as this value represents the typical limit of detection. In an effort to minimize overestimation, given that infertility is not a rare health outcome, we derived the RR for each OR across the percentile distribution of both phthalates using a formula previously applied in our US study [59].

We then applied data on TTP from the LIFE Study in order to interpret the reduced fecundity in terms of increases in TTP greater than 12 months. In doing so, we modeled shifts in the TTP by multiplying the number of months to conception by the inverse of the RR of infertility across the distribution of percentiles of exposure. Thus, we calculated the percentage of the population with a shift in TTP above 12 months to be the incremental prevalence of infertility attributable to either MBZP or MnBP exposure. In accordance with our previous study, this incremental prevalence was applied to 88% of the population, which is the percentage of the population that conceived in less than 12 months.

We then obtained data on contraceptive use of any form among Canadian women [60] and the number of Canadian women either married or living in common law in 2011 [61] with the assumption that 2011 estimates are a close approximation of 2010 estimates. To determine the AF of ART services due to infertility associated with phthalate exposure, we accounted for both the baseline prevalence of infertility in Canada [58] as well as the incremental prevalence of infertility attributable to phthalate exposure. This AF was then multiplied by the number of women 20–39 years old who were either married or living in common [61] law and not using contraception of any method to estimate the number of couples who sought ART in the setting of phthalate-attributable infertility.

Economic Evaluation

The cost estimate was obtained from a review that investigated the direct cost of ART treatment until pregnancy was achieved in several countries, including both the US and Canada [62]. The review estimates the direct cost of a standard IVF cycle in Canada at $8,500 (2006 USD), which was corrected to 2010 CAD using the US CPI [11] and Canadian PPP [7].

3.2. Methods for Female Reproductive Disorders

3.2.1. Modeling DDE-Attributable Uterine Fibroids

Exposure–Response Relationship

We applied lipid-adjusted plasma DDE levels, which were subdivided into quintiles, for women 20–39 years of age in the 2007–2009 survey cycle of CHMS. Data for the exposure–response relationship was obtained from a study that was applied in our US paper, as Canadian data was unavailable. The study identified a significantly increased OR (1.37) of uterine fibroids in the setting of elevated DDE levels [63]. The OR was applied across the percentiles of the exposure distribution, using the 10th percentile applied in the US study (76.5 ug/L) as a conservative reference. To quantify the number of DDE-attributable

cases of uterine fibroids, the summation of ORs for each percentile was then multiplied by the incident rate of uterine fibroids in Canada. As the Canadian incidence of uterine fibroids was unavailable in the literature, we derived its value by multiplying the US incidence [64] by the ratio of the US to Canadian prevalence [65]. Census data for the population of Canadian women 15–54 years of age in 2010 was obtained from Statistics Canada [4].

Economic Evaluation

Cost estimates were obtained from a systematic review of the costs of uterine fibroid tumors [66], which identified a Canadian analysis that reported a direct cost of $1,688.18 (2013 USD) per myomectomy [67]. We updated the cost estimate to the 2010 Canadian dollar using the US Consumer Price Index [11] and the Canadian PPP [7].

3.2.2. Modeling Phthalate-Attributable Endometriosis

Exposure–Response Relationship

We applied urinary levels of phthalate metabolites (MEHHP, MEHP, MEOHP) that together constitute DEHP for women 20–39 years of age in the 2009–2011 survey cycle of CHMS. However, CHMS did not include data regarding levels of MECPP, an additional metabolite in the calculation of DEHP. In order to derive total DEHP levels in this Canadian population estimate, we obtained MECPP, MEOHP, MEHP and MEHHP levels for women 20–39 years of age from NHANES in 2009–2010. We then multiplied the US MECPP value by the ratio of the sum of the three other phthalates in the Canadian and US datasets across each percentile in order to arrive at an interpolated MECPP value in Canadian women.

Data for the exposure–response relationship was obtained from a study that was previously applied in our US paper, as Canadian data was unavailable. The study identified a significantly increased OR (1.35) of endometriosis in the setting of elevated DEHP levels [68]. To extrapolate the burden of endometriosis attributable to DEHP exposure, we first exponentiated the OR identified by Buck Louis and colleagues across each percentile of exposure in log unit form. Each OR was then multiplied by the incidence of endometriosis in the US [69], as Canadian data was unavailable in the literature, to calculate the exposure rate. Finally, the unexposed rate was subtracted from the exposed rate in order to arrive at the DEHP-attributable incremental rate of endometriosis. The incremental rate was then multiplied by the pertinent Canadian population estimate in 2010 [4] to quantify the number of endometriosis cases attributable to DEHP exposure.

Economic Evaluation

Estimates for direct costs associated with endometriosis were obtained from a Canadian study [70], while indirect cost estimates using lost DALYs due to endometriosis (valued at $ 50,000 USD) over 10 years were taken from a study previously applied in our US study [71]. The direct cost estimate was corrected from the 2009 to 2010 Canadian dollar using the Canadian Consumer Price Index [24], while the indirect cost estimate was corrected from the USD to CAD dollar using the Canadian currency exchange rate for 2010 [7]. The direct and indirect costs were then combined to determine the total costs of DEHP-attributable endometriosis.

Results

4. Neurodevelopmental Deficit and Disability Burden

4.1. PBDE-Attributable IQ Deficits and Intellectual Disability

Our base case estimate revealed that in utero exposure to PBDE-47 was associated with 374,000 lost IQ points and 1,600 ID cases in the 2010 birth cohort, resulting in annual

costs of $8.8 billion and $2.6 billion CAD, respectively. In our sensitivity analysis, we found that IQ point loss ranged from 791,000 to 925,000, which was associated with 3700–4500 ID cases and $18.6–21.8 billion and $6.0–7.4 billion CAD, respectively.

4.2. OP-Associated IQ Loss and Intellectual Disability

The base case estimate identified 153,000 lost IQ points and 377 ID cases attributable to in utero OP exposure, leading to costs of $3.6 billion and $619 million CAD, respectively. In the sensitivity analysis, the low-end estimate identified 50,000 lost IQ points, 111 ID cases and associated costs of $1.2 billion and $182 million CAD, respectively. The high-end estimate identified 201,000 lost IQ points, 522 ID cases and associated costs of $4.7 billion and $858 million CAD, respectively.

4.3. Autism Attributable to Multiple Exposures

The 10th percentile of exposure was applied as the reference level, above which all exposure values were log-transformed. The point increase in the Social Responsiveness Scale (SRS) T-score values ranged from 2.08 to 3.42. An increase above the SRS reference level (75) was associated with autism between 0.622–1.088% in males and 0.173–0.314% in females, applying US normative data as previously done in our US study [72]. Based on these calculations, the AF was then estimated to be 95.6% in males and 27.0% in females. To account for the likelihood that EDCs other than phthalates contributed to autism, we applied a base case estimate of the AF at 5% with sensitivity analyses (SA) of 2% and 10%, as in our prior US and EU studies.

Finally, we applied the prevalence of childhood autism in Canada among males and females and estimated that phthalate exposure was associated with 118 (SA: 47–236) additional autism cases in males and 28 (SA: 11–56) additional autism cases in females. The overall lifetime cost of phthalate-associated autism in males and females together was estimated at $233 million CAD ($93–466 million).

4.4. ADHD Attributable to Multiple Exposures

In the main analysis, the relative risk of ADHD associated with OP exposure was 1.08 and 1.17 in the two highest percentiles of exposure. The corresponding overall AF was 2.81%. By applying this AF, the prevalence of childhood ADHD in Canada (2.14%), and the number of 12-year-old Canadian children, an additional 180 cases of OP-associated ADHD were identified. The associated cost, aggregated over 10 years, was estimated at $34.8 million CAD (range: $28.4-41.3 million).

In the sensitivity analysis, the relative risk of ADHD associated with PBDE exposure was 1.12 and 1.41 in the highest two percentiles of exposure. The AF was estimated at 7.41%, which yielded 329 attributable ADHD cases and associated costs of $63.6 million CAD (range: $51.8-75.4 million).

5. Metabolic Disorders

5.1. DDE-Attributable Adult Diabetes

Plasma DDE levels ranged from 0.38 ug/L in the 11th–25th percentile to 3.89 ug/L in the 90th–99th percentile. In the base case estimate, we found that an additional 14.0 newly incident diabetes cases per 10,000 person-years in the top 25% were attributable to DDE exposure. After accounting for prior diabetes cases, this incident rate translates into an additional 3270 cases of adult diabetes per year with associated costs of $385.2 million CAD annually aggregated over 15 years. In the sensitivity analysis, we found an additional 15.5 newly incident diabetes per 1,000 person years in the top 25%, which translated into 36,209 cases of DDE-attributable diabetes per year with associated costs of $4.3 billion CAD aggregated over 15 years.

5.2. DDE-Attributable Childhood Overweight

Plasma DDE levels ranged from 35.94 to 478.63 ug/kg (lipid-adjusted) across the percentile distribution. In the main analysis, the increments in the change in weight-for-age Z-scores ranged from 0.003 to 0.07 for the exposed distribution of percentiles. These increments translate into 0.10–2.29% increases in rapid weight gain in infancy. Among 72,900 cases of overweight 10-year-old children in Canada, 0.37% were attributable to DDE-mediated increases in rapid infant weight gain. These estimates translate into a total cost of $2.5 million CAD. In the sensitivity analysis, we found that incremental increases in the change in weight-for-age Z-scores ranged from 0.14 to 1.12, which translate into 0.43% to 3.68% increases in rapid infant weight gain. Our estimates indicate that 0.44% of the total overweight cases among 10-year-old Canadian children were associated with DDE-attributable increases in rapid infant weight gain with costs of $6.9 million CAD.

5.3. Phthalate-Attributable Adult Obesity

The median urinary phthalate level in the 75th and 90th percentiles exceeded the reference level [35]. As such, the increment in weight gain was only estimated for the two highest percentiles of phthalate exposure. An annual increment in weight gain of 0.07 and 0.09 kg/year was applied for the two highest percentiles, respectively, resulting in 2093 cases of phthalate-attributable obesity and associated total costs of $684 million CAD.

5.4. Phthalate-Attributable Adult Diabetes

Total phthalate levels, stratified into quintiles, ranged from 101.29–700.07 nmol/L. We estimated an OR of 1.09 in the highest percentile of exposure. After accounting for pre-existing cases of type II diabetes mellitus, we identified an additional 225 cases attributable to phthalate exposure with an associated cost of $25.8 million CAD aggregated across 15 years with 3% discounting.

5.5. BPA-Attributable Childhood Obesity

In the main analysis using a linear increment of 0.28 [41], we identified BMI z-score increments ranging from 0.01 to 0.18 across percentiles of exposure above the reference level. Corresponding increases in the childhood obesity prevalence attributable to BPA ranged between 0.0012 and 0.0220, which resulted in an additional 1023 cases. Of these, 511 cases were estimated to remain obese into adulthood. The total cost of childhood obesity attributable to prenatal BPA exposure was estimated at $59 million CAD, which is comprised of $4.7 million in direct costs and $54.3 million in indirect costs.

In the sensitivity analysis using an increment of 0.20 [43], BMI z-score increments ranged from 0.008 to 0.131 across percentiles of exposure above the reference level. Corresponding increases in the childhood obesity prevalence ranged between 0.0009 and 0.015, which resulted in an additional 711 cases, of which 355 cases would persist into adulthood. The total cost was estimated at $41 million CAD, which is comprised of $3.3 million in direct costs and $37.8 million in indirect costs.

6. Reproductive Disorders

6.1. Male Reproductive Disorders

6.1.1. PBDE-Attributable Testicular Cancer

As in our US study, the base case estimate of PBDE-AFs of testicular cancer cases was 42.9%, while our sensitivity analysis revealed a range of 13.0% to 57.4%. Exposure to PBDE-47 resulted in 316 cases of testicular cancer in the base case estimate, with the sensitivity analysis identifying a range between 96 and 423 cases. We found that the base case estimate 5-year direct costs of PBDE-attributable testicular cancer in Canada were $7.3 million CAD (sensitivity analysis: $2.2–9.8 million).

6.1.2. PBDE-Attributable Cryptorchidism

Exposure levels, stratified by quintiles, ranged from 2.56–50.62 ug/kg lipid. The estimated ORs for the 75th and 90th percentiles were 1.53 and 3.14, respectively. When these ORs were applied to our calculation of the AF of PBDE-attributable cryptorchidism cases, we identified 567 cases with an estimated cost of $5.8 million CAD.

6.1.3. Phthalate-Associated Reductions in Testosterone And All-Cause Mortality

Exposure levels, stratified by quintiles, ranged between 0.04–0.32 μmol/L and 0.02-0.16 μmol/L for DEHP and MnBP, respectively. Phthalate-associated reductions in testosterone ranged between 6.5–34.2% and 5.5–55.4% across percentiles for DEHP and MnBP, respectively. When accounting for concurrent DEHP and MnBP exposure, the mean reduction in testosterone ranged between 1.64 and 12.20 μmol/L, which corresponds with an increased relative risk of all-cause mortality between 1.07 and 1.61 across exposure percentiles. These phthalate-associated reductions in testosterone accounted for 3,385 deaths and $1.8 billion CAD in lost economic productivity.

6.1.4. Phthalate-Associated Increases in the Use of Assisted Reproductive Technology

By applying the ORs identified in the LIFE Study to the CHMS exposure groups, we found that increased TTP was associated with ORs of 0.51–0.68 for MNBP and 0.65–0.82 for MBZP. Next, we determined that phthalate-attributable infertility above the baseline prevalence of infertility in Canada was in the range of 3–5% for MNBP and 3–4% for MBZP. The corresponding overall increments in infertility attributable to either MBZP or MNBP across the Canadian population were 3.2% and 3.7%, respectively. After accounting for overlap, we calculated an AF of 5.99%

We then estimated that 2.4 million Canadian women between the ages of 20–39 years of age were both (1) either married or in common law (based on 2011 data as 2010 data was unavailable) [4], and (2) not using any method of contraception (most recent data from 2002) [60]. We applied this value to the AF while taking into account that only 3.8% of Canadian couples experiencing infertility sought out infertility services (Gunby, n.d., Bushnik et al., 2012). This calculation revealed that phthalate-attributable infertility was associated with 1,395 infertility treatments, which cost a total of $17 million CAD.

6.2. Female Reproductive Disorders

6.2.1. DDE-Attributable Uterine Fibroids

The estimated ORs for percentiles of exposure ranged from 1.003 to 1.28. These ORs were then applied to the incident rate of uterine fibroids in Canada, which we calculated to be 0.012. We estimated that 2254 Canadian women between the ages of 20 and 39 years old were found to have uterine fibroids attributable to DDE exposure, resulting in $4.2 million CAD in direct costs.

6.2.2. Phthalate-Attributable Endometriosis

The incremental incidence of endometriosis ranged from 2.0/1000 to 3.0/1000, which amounted to a total of 10,151 cases of DEHP-attributable endometriosis. The total costs across 10 years were estimated at $5.7 billion CAD ($11.9 million in direct costs, $5.6 billion in indirect costs)

Table S1. List of Endocrine-Disrupting Chemicals in the Analysis.

Endocrine Disrupting Chemicals	Major Metabolites Examined	Matrix Used in CHMS
Polybrominated diphenyl ethers (PBDE)	PBDE-47	Plasma (lipid adjusted)
Organophosphate pesticides (OP)	Dimethylphosphate (DMP)<br>Dimethylthiophosphate (DMTP)<br>Diethylphosphate (DEP)	Urine

Dichlorodiphenyldichloroethylene (DDE)		Plasma (± lipid adjusted)
Phthalates	Monoethyl phthalate (MEP)<br>Monobutyl phthalate (MBP)<br>Mono-isobutyl phthalate (MiBP)<br>Mono(2-ethylhexyl) phthalate (MEHP)<br>Monobenzyl phthalate (MBzP)<br>Mono(2-ethyl-5-oxohexyl) phthalate (MEOHP)<br>Mono(2-ethyl-5-hydroxyhexyl) phthalate (MEHHP)<br>Mono(2-ethyl-5-carboxypentyl) phthalate (MECPP)	Urine
Di-2-ethylhexylphthalate (DEHP)	Mono(2-ethylhexyl) phthalate (MEHP)<br>Mono(2-ethyl-5-carboxypentyl) phthalate (MECPP)<br>Mono(2-ethyl-5-hydroxyhexyl) phthalate (MEHHP)<br>Mono(2-ethyl-5-oxohexyl) phthalate (MEOHP)	Urine
Bisphenol A		Urine (creatinine-corrected)

Table S2. Summary of Epidemiologic Studies Applied in this Analysis.

	**Exposure**	**Outcome**	**Epidemiologic Studies**	**Risk Estimates (base case)**	**Risk Estimates (sensitivity analyses & alternative scenarios)**
**1**	PBDE	IQ Loss and Intellectual Disability	Chen et al., 2014 (base case) Eskenazi et al., 2013 (low-end scenario) Herbstman et al. 2010 (high-end scenario)	4·5-point IQ loss (95% CI: 8·8, 0·1) per 10- fold increase in prenatal PBDE47 (6·4 ng/g reference level)	Low end: 4·7-point IQ loss (95% CI: 9·4, 0·1) per 10-fold increase in prenatal PBDE47 (2·82 ng/g reference level) High end: 5·5-point IQ loss (range 5·5 to 8·8) per 10-fold increase in prenatal PBDE47 (2·82 ng/g reference level)
**2**	OP	IQ Loss and Intellectual Disability	Bellinger, 2012 (base case) Engel et al., 2011 (low-end scenario) Bouchard et al., 2011 (high-end scenario)	4·25-point IQ loss (weighted estimate) for a 10-fold increase in prenatal exposure (65 nmol/l reference level)	Low end: 1·39-point IQ loss (95% CI: 4·5, 1·77);for a 10-fold increase in prenatal exposure (65 nmol/l reference level) High end: 5·6-point IQ loss (95% CI: 2·2-9·0) for a 10-fold increase in prenatal exposure (65 nmol/l reference level)
**3**	DDE	Childhood obesity	Iszatt et al., 2015 (base case) Valvi et al., 2012 (sensitivity analyses)	Identified 0·12 change (95% CI: 0·03, 0·22)in weight-for-age Z score across the interquartile range	Identified a RR of 1·67 (95% CI: 1·10, 2·55) and of 1.28 (95% CI: 0·81, 2·03) in the second and third tertile of DDE exposure, respectively.
**4**	DDE	Adult diabetes	Wu et al., 2013 (base case) Turyk et al., 2009 (sensitivity analyses)	Reported pooled OR of 1·25 (95% CI: 0·94, 1·66) in the highest quartile of DDE exposure	Identified annual increments in newly incident cases reported for specific range of exposure (incidence/1,000 person-years of 17·1 in the highest tertile of DDE exposure), with incidence rate

					ratio (IRR) of 7·1 (95% CI: 1·6, 31·9)
**5**	DEHP	Adult obesity	Song et al., 2014 (base case)	Identified annual weight change rate by quartiles of urinary phthalate metabolite concentrations : 0·09 (95% CI: -0·07, 0·24);, 0·21 (95% CI: 0·06, 0·37); and 0·17 (95% CI: 0·02, 0·33) kg/year in the 2nd, 3rd and 4th quartile.	
**6**	DEHP	Adult diabetes	Sun et al., 2014 (base case)	Reported OR of 2·14 (95% CI: 1·19, 3·85)for incident cases of type 2 diabetes by quartiles of urinary concentrations of phthalate metabolites	
**7**	BPA	Childhood obesity	Valvi et al., 2013 (base case), Vafeiadi et al., 2016 (sensitivity analyses)	Identified 0·28 (95% CI: -0·06, 0·63) linear increment in BMI z-score at 4 years of age associated with BPA exposure	Identified 0.20 increment in BMI z-score at 4 years of age associated with BPA exposure
**8**	PBDE	Testicular cancer	Hardell et al., 2006	Reported OR of 2·5 (95% CI: 1·02, 6·0) for testis cancer in association with higher maternal serum levels of PBDEs	
**9**	PBDE	Cryptorchidism	Main et al., 2007 Mannetje et al., 2012	Main et al: Identified association between infant exposure to PBDE (assessed by measuring the concentration of PBDEs in breast milk) and cryptorchidism:	

				median levels 4.16 (1.39–51.62) vs. 3.16 (1.08– 21.47) ng/g fat in cryptorchid boys vs controls; $p < 0.007$. Mannetje et al: Reported serum/milk ratios of POPs in paired human samples, with an average ratio of 0·8	
**10**	Benzyl (MBZP) and Butylphthalates (MBP)	Male infertility resulting in increased use of Assisted Reproductive Technology (ART)	Buck Luis et al., 2014	In a longitudinal study (the LIFE Study), men's urinary concentrations of monomethyl, mono-n-butyl, and monobenzyl phthalates were associated with a longer time-to-pregnancy (TTP) with fecundability OR of 0·80 (95% CI, 0·70, 0·93); 0·82 (95% CI, 0·70, 0·97), and 0·77 (95% CI, 0·65 0·92), respectively).	
**11**	Phthalates (MBP and DEHP)	Low testosterone resulting in increased early all-cause mortality	Araujo et al., 2011	Reported RR of 1·35 (95% CI: 1·13, 1·62) for increased all-cause mortality in association with lower testosterone levels	
**12**	Multiple Exposures (OP and PBDE)	ADHD	Bouchard et al. 2010 (OPs, base case) Gascon et al., 2011 (PBDE-47, sensitivity analyses)	Reported OR of 1·35 (95% CI: 1·10, 1·67) for any ADHD subtype for a 10-fold increase in total urinary dialkyl phosphate metabolites	Reported OR of 1·80 (95% CI:1·0, 3·2) for attention deficit symptoms in relation to PBDE- 47 exposure
**13**	Multiple Exposures (phthalates)	Autism	Miodovnik et al., 2011	Identified 1·53 increment (95% CI: 0·25, 2·8) in the	

				social responsiveness score (SRS) per log unit increase of phthalate metabolites	
**14**	DDE	Fibroids	Trabert et al., 2015	Evaluated the association between persistent organic pollutants (POPs) and uterine fibroids. Among POPs, serum DDE levels were associated with fibroids, with OR of 1·37 (95% CI: 1·05-1·80) per 1-SD increase in log-transformed DDE	
**15**	DEHP	Endometriosis	Buck Luis et al., 2013	Identified an approximately twofold increase in the odds of an endometriosis diagnosis for 1 SD increase in the concentration of selected phthalate metabolites: MECPP 2·92 (95% CI: 1·46, 5·84); MEHHP 2·20 (95% CI: 1·23, 3·94); MEOHP 2·33 (95% CI: 1·26, 4·29) MEHP 2·59 (95% CI: 1·17, 5·75)	

Table S3. PBDE-Attributable IQ Loss, Intellectual Disability and Associated Costs (Canadian Children Born in 2010).

Expert Panel Evaluation of Epidemiologic Evidence	Moderate-to-high					
Expert Panel Evaluation of Toxicologic Evidence	Strong					
Probability of Causation	70–100%					
Percentile of Exposure	0–9	10–24	25–49	50–74	75–89	>90
Percentile Assumed	0	10	25	50	75	90
Maternal Plasma PBDE, μg/kg lipid (Base Case)	0	2.56	4.37	10.98	19.37	50.62
IQ loss (Base Case)	0	0	0	1.05	2.16	4.04

IQ loss (High Estimate)	0	0	1.05	3.25	4.60	6.90
Births	37,721	56,581	94,303	94,303	56,561	37,721
IQ points lost (Base Case)	0	0	0	99,480	122,458	152,455
IQ points lost (High Estimate)	0	0	98,667	306,197	260,437	260,178
Lost Economic Productivity (Base Case)			$8.8 billion			
Lost Economic Productivity (High Estimate)			$21.8 billion			
Attributable Intellectual Disability (Base Case)			1610 cases			
Attributable Intellectual Disability (High Estimate)			4491 cases			
Cost of Intellectual Disability (Base Case)			$2.6 billion			
Cost of Intellectual Disability (High Estimate)			$7.4 billion			
Total Costs (Base Case)			$11.5 billion			
Total Costs (High Estimate)			$29.2 billion			

Table S4. OP-Attributable IQ Loss, Intellectual Disability and Associated Costs (Canadian Children Born in 2010).

Expert Panel Evaluation of Epidemiologic Evidence	Moderate-to-high					
Expert Panel Evaluation of Toxicologic Evidence	Strong					
Probability of Causation	70–100%					
Percentile of Exposure	0–9	10–24	25–49	50–74	75–89	>90
Percentile Assumed	0	10	25	50	75	90
Urinary Total Dialkylphosphate, nmol/L (Base Case)	0	0	0	37.43	119.37	234.88
IQ loss (Base Case)	0	0	0	0	1.12	2.37
IQ loss (Low End Estimate)	0	0	0	0	0.37	0.78
IQ loss (High)	0	0	0	0	1.48	3.12
Births	0	56,581	94,303	94,303	56,581	37,721
IQ points lost (Base Case)	0	0	0	0	63,477	89,445
IQ points lost (Low)	0	0	0	0	20,761	29,254
IQ points lost (High)	0	0	0	0	83,640	117,857
Lost Economic Productivity (Base Case)	$3.6 billion					
Lost Economic Productivity (Low)	$1.2 billion					
Lost Economic Productivity (High)	$4.7 billion					
Attributable Intellectual Disability (Base Case)	377 cases					
Attributable Intellectual Disability (Low)	111 cases					
Attributable Intellectual Disability (High)	522 cases					
Cost of Intellectual Disability (Base Case)	$619.3 million					
Cost of Intellectual Disability (Low)	$182.2 million					
Cost of Intellectual Disability (High)	$857.6 million					
Total Costs (Base Case)	$4.2 billion					
Total Costs (Low)	$1.4 billion					
Total Costs (High)	$5.6 billion					

Table S5. DDE-Attributable Childhood Overweight and Associated Costs (10-Year-Old Canadian Children in 2010).

Expert Panel Evaluation of Epidemiologic Evidence	Moderate					
Expert Panel Evaluation of Toxicologic Evidence	Moderate					
Probability of Causation	40–69%					
Percentile of Exposure	0–9	10–24	25–49	50–74	75–89	>90
Percentile Assumed	0	10	25	50	75	90
Maternal plasma DDE levels (ug/kg lipid)	0	35.94	49.64	78.19	140.55	478.63
Increment in Change in Weight for Age Z-Score (Main Estimate)	0	0	0.003	0.006	0.01	0.07
Relative Risk of Rapid Infant Weight Gain (Sensitivity Analysis)	1.0	1.0	0.14	0.34	0.59	1.12
Attributable Increment in Rapid Weight Gain (Main Estimate)	0	0	0.10%	0.20%	0.43%	2.29%
Attributable Increment in Rapid Weight Gain (Sensitivity Analysis)	0	0	0.43%	1.05%	1.88%	3.68%
Attributable Fraction of Overweight at Age 10 (Main Estimate)	0.37%					
Attributable Fraction of Overweight at Age 10 (Sensitivity Analysis)	1.02%					
Attributable Cases of Overweight (Main Estimate)	114 cases					
Attributable Cases of Overweight (Sensitivity Analysis)	318 cases					
Costs of Attributable Overweight (Main Estimate)	$2.5 million					
Costs of Attributable Overweight (Sensitivity Analysis)	$6.9 million					

Table S6. DDE-Attributable Adult Diabetes and Associated Costs (Canadian Adults 40-59 Years of Age in 2010).

Expert Panel Evaluation of Epidemiologic Evidence	Low					
Expert Panel Evaluation of Toxicologic Evidence	Moderate					
Probability of Causation	20–39%					
Percentile of Exposure	0–9	10–24	25–49	50–74	75–89	>90
Percentile Assumed	0	10	25	50	75	90
Plasma DDE levels (ug/L)	0	0.38	0.54	0.86	1.89	3.89
Increment in diabetes cases applied annually (Main Estimate)	0	0	0	0	0.0014	0.0014
Increment in diabetes cases applied annually (Sensitivity Analysis)	0	0	0	0	0.0155	0.0155
Annual attributable cases (Main Estimate)	0	0	0	0	2079	1386
Annual attributable cases (Sensitivity Analysis)	0	0	0	0	23,014	15,343
Annual attributable cases accounting for preexistent diabetes (Main Estimate)	3270 cases					
Annual attributable cases accounting for preexistent diabetes (Sensitivity Analysis)	36,209 cases					
Annual direct cost for attributable cases (Main Estimate)	$385.2 million					
Annual direct cost for attributable cases (Sensitivity Analysis)	$4.3 billion					

Table S7. Phthalate-Attributable Adult Obesity and Associated Costs (Canadian Women 40-59 Years Old in 2010).

Expert Panel Evaluation of Epidemiologic Evidence	Low					
Expert Panel Evaluation of Toxicologic Evidence	Strong					
Probability of Causation	40–69%					
Percentile of Exposure	0–9	10–24	25–49	50–74	75–89	>90
Percentile Assumed	0	10	25	50	75	90
Midpoint Urinary DEHP, nmol/L	50.65	113.13	170.65	310.13	552.01	954.32
Annual weight gain (kg/year)	0	0	0	0	0.071	0.086
Attributable cases of obese females	2,093					
Attributable direct costs	$249,147					
Attributable indirect costs	$684.6 million					
Attributable total costs	$684.8 million					

Table S8. Phthalate-Attributable Adult Diabetes and Associated Costs (Canadian Women 40-59 Years Old in 2010).

Expert Panel Evaluation of Epidemiologic Evidence	Low					
Expert Panel Evaluation of Toxicologic Evidence	Strong					
Probability of Causation	40–69%					
Percentile of Exposure	0–9	10–24	25–49	50–74	75–89	>90
Percentile Assumed	0	10	25	50	75	90
Urinary DEHP, nmol/L	0	101.29	125.97	216.33	404.94	700.07
Odds ratio for newly incident diabetes	1	1	1	1	1	1.09
Increment in newly incident diabetes	0	0	0	0	0	0.0005
Annual attributable cases	239 cases					
Annual attributable cases accounting for preexistent diabetes	225 cases					
Annual direct cost for attributable cases	$25.8 million					

Table S9. Main Estimate: BPA-Attributable Childhood Obesity and Associated Costs (4-Year-Old Canadian Children, 2010).

Expert Panel Evaluation of Epidemiologic Evidence	Very Low to Low					
Expert Panel Evaluation of Toxicologic Evidence	High					
Probability of Causation	20–69%					
Percentile of Exposure	0–9	10–24	25–49	50–74	75–89	>90
Percentile Assumed	0	10	25	50	75	90
Urinary BPA levels (ug/g creatinine)	0	0.48	0.84	1.1	2.85	4.5
Increment in BMI Z-Score	0	0	0	0.01	0.13	0.18
Increment in obesity prevalence at age 4	0	0	0	0.001	0.01	0.02
Attributable cases of childhood obesity	0	0	0	114.23	826.38	82.79
Attributable cases of adult obesity	0	0	0	57.11	413.19	41.40
Attributable direct costs	$4,705,820					
Attributable indirect costs	$54,318,277					
Attributable total costs	$59,024,097					

Table S10. Sensitivity Analysis: BPA-Attributable Childhood Obesity and Associated Costs (4-Year-Old Canadian Children, 2010).

Expert Panel Evaluation of Epidemiologic Evidence	Very Low to Low					
Expert Panel Evaluation of Toxicologic Evidence	High					
Probability of Causation	20-–69%					
Percentile of Exposure	0–9	10–24	25–49	50–74	75–89	>90
Percentile Assumed	0	10	25	50	75	90
Urinary BPA levels (ug/g creatinine)	0	0.48	0.84	1.1	2.85	4.5
Increment in BMI Z-Score	0	0	0	0.01	0.09	0.13
Increment in obesity prevalence at age 4	0	0	0	0.001	0.01	0.02
Attributable cases of childhood obesity	0	0	0	81.37	573.23	56.72
Attributable cases of adult obesity	0	0	0	40.68	286.62	28.36
Attributable direct costs	$3,270,839					
Attributable indirect costs	$37,754,599					
Attributable total costs	$41,025,439					

Table S11. PBDE-Attributable Testicular Cancer and Associated Costs (Canadian Men Aged 20-79 in 2010).

**Expert Panel Evaluation of Epidemiologic Evidence**	**Very Low**
Expert Panel Evaluation of Toxicologic Evidence	Weak
Probability of Causation	0–19%
Exposure Prevalence (Sensitivity Analysis)	50% (10%, 90%)
Odds Ratio	1.50
Attributable Fraction (Sensitivity Analysis)	42·9% (13·0–57·4%)
Annual incidence	6/100,000
Annual newly incident cases	737
Attributable cases (Sensitivity Analysis)	316 (96–423)
Attributable costs (sensitivity analysis)	$7.3 million ($2.2–$9.8million)

Table S12. PBDE-Attributable Cryptorchidism and Associated Costs (All Male Newborns in 2010).

Expert Panel Evaluation of Epidemiologic Evidence	Low					
Expert Panel Evaluation of Toxicologic Evidence	Strong					
Probability of Causation	40–69%					
Percentile of Exposure	0–9	10–24	25–49	50–74	75–89	>90
Percentile Assumed	0	10	25	50	75	90
Plasma Levels PBDE-47 (ug/kg, lipid-adjusted)	**0**	2.56	4.37	10.98	19.37	50.62
Estimated Breast Milk PBDE-47 (Base Case)	0.00	3.20	5.46	13.73	24.21	63.28
Odds of Cryptorchidism	1.0	1.0	1.0	1.0	1.53	3.14
Attributable Increment in Cryptorchidism	567 cases					
Attributable Costs of Cryptorchidism	$5.8 million					

Table S13. Phthalate-Attributable Male Infertility Resulting in Use of Assisted Reproductive Technology and Associated Costs (Canadian Men 20–39 Years Old in 2011).

Expert Panel Evaluation of Epidemiologic Evidence	Low					
Expert Panel Evaluation of Toxicologic Evidence	Strong					
Probability of Causation	40–69%					
Percentile of Exposure	0–9	10–24	25–49	50–74	75–89	>90
Percentile Assumed	0	10	25	50	75	90
Urinary MBZP (ug/L)	0	2	5.28	7.6	16	27.84
MBzP-exposed fecundity odds ratio	1.00	0.82	0.75	0.73	0.69	0.65
Urinary MBP (ug/L)	0	5.9	15.90	23	44	73.61
MBP-exposed fecundity odds ratio	1.00	0.68	0.61	0.58	0.54	0.51
Rate of infertility across entire population of couples attributable to MBzP	3.2%					
Rate of infertility across entire population of couples attributable to MBP	3.7%					
Total rate of infertility across entire population attributable to MBP +/or MBzP (accounting for double counting)	6.78%					
Women (20-39) living in common law or married	2,386,365					
Women (20-39) not using contraception living in common law or married	620,455					
Attributable infertility seeking medical care	1395					
Cost of assisted reproductive technology attributable to phthalate exposure	$17 million					

Table S14. Phthalate-Attributable Decreased in Testosterone Resulting in Increased Early All-Cause Mortality and Associated Costs (Canadian Men 40-59 Years old in 2010).

Expert Panel Evaluation of Epidemiologic Evidence	Low					
Expert Panel Evaluation of Toxicologic Evidence	Strong					
Probability of Causation	40-69%					
Percentile of Exposure	0-9	10-24	25-49	50-74	75-89	>90
Percentile Assumed	0	10	25	50	75	90
Urinary monobutylphthalate (MBP), µmol/L	0	0.02	0.03	0.08	0.13	0.16
Urinary DEHP metabolites, µmol/L	0	0.04	0.09	0.13	0.23	0.32
MBP related change in testosterone (10th percentile as reference level)	0	0	-0.06	-0.23	-0.44	-0.55
DEHP related changes in testosterone (10th percentile as reference level)	0	0	-0.07	-0.11	-0.23	-0.34
Change in additive mean T (µmol/l)	0	0	-1.64	-4.53	-9.18	-12.20
Mortality RR (assuming 1·35 RR per 7.72 nmol/l increment in testosterone)	0	1	1.07	1.19	1.43	1.61
Deaths, 55-64 year old men (baseline)	17,869					
Incremental deaths resulting from phthalate-attributable testosterone	3,385					
Lost economic productivity	$1.8 billion					

Table S15. Main Estimate: OP-Attributable ADHD and Associated Costs (12-Year-Old Canadian Children in 2010).

Percentile of Exposure	0-9	10-24	25-49	50-74	75-89	>90
Percentile Assumed	0	10	25	50	75	90
Total urinary alkyl phosphate, nmol/L	0	0	0	37.43	119.37	234.88
Estimated relative risk, ADHD	1.0	1.0	1.0	1.0	1.08	1.17
Attributable Fraction	2.81%					
Prevalence among 12 year old children, ADHD	2.14%					
Attributable ADHD Cases after Accounting for Coexistent Intellectual Disability	180					
Cost of Attributable Cases	$34.8 million					

Table S16. Sensitivity Analysis: PBDE-Attributable ADHD and Associated Costs (12-Year-Old Canadian Children in 2010).

Odds Ratio, ADHD	1.80
Attributable Fraction	7.4%
Prevalence among 12 year old children, ADHD	2.14%
Attributable ADHD cases after accounting for coexistent intellectual disability	329
Cost of Attributable Cases	$63.6 million

Table S17. Estimates of Phthalate-Attributable Fractions for Autism (8-year-old Canadian Children in 2010).

Percentile of Exposure	0-9	10-24	25-49	50-74	75-89	>90
Percentile Assumed	0	10	25	50	75	90
Low molecular weight (LMW) phthalates, ug/L	0	378.70	605.10	1211.85	2046.80	4529.98
Increase in Responsiveness Score, assuming 10th percentile reference level and 1·53 increase/log (LMW)	0	2.08	2.54	2.89	3.42	2.08
Increase in SRS>75, Assuming Mean and SD from Normative Data (Males)	0	0.62%	0.78%	0.90%	1.09%	0.62%
Increase in SRS>75, Assuming Mean and SD from Normative Data (Females)	0	0.17%	0.22%	0.26%	0.31%	0.17%
Overall Attributable Fraction (Males, Females)				(95.6%, 27.0%)		

Table S18. Estimates of Endocrine Disruptor Attributable Autism and Costs (8-year-old Canadian Children in 2010).

Expert Panel Evaluation of Epidemiologic Evidence	Low
Expert Panel Evaluation of Toxicologic Evidence	Moderate
Probability of Causation	20-39%
Attributable Fraction (Sensitivity Analysis)	5% (2-10%)
Prevalence of Autism	15.2 per 1,000
Autism Cases, 8-Year Old Children	5,680
Attributable Autism Cases after Accounting for Coexistent Intellectual Disability, 2010 (Sensitivity Analysis)	146 (58 - 292)
Attributable Lifetime Autism Costs, 2010 (Sensitivity Analysis)	$233 million ($93 million – $466 million)

Table S19. DDE-Attributable Fibroids and Associated Costs (Canadian Women 15-54 Years of Age in 2010).

Expert Panel Evaluation of Epidemiologic Evidence	Low					
Expert Panel Evaluation of Toxicologic Evidence	Moderate					
Probability of Causation	20-39%					
Percentile of Exposure	0-9	10-24	25-49	50-74	75-89	>90
Percentile Assumed	0	10	25	50	75	90
Plasma DDE, ug/kg lipid-adjusted	0	35.94	49.64	78.19	140.55	478.63
Odds Ratio	0	1	1	1.003	1.09	1.28
Unexposed Incidence	0.012					
Incremental Incidence	0	0	0	0	0.001	0.003
Attributable Cases	2,254					
Attributable Costs	$4.2 million					

Table S20. DEHP-Attributable Endometriosis and Associated Costs (Canadian Women 20-39 Years of Age in 2010).

Expert Panel Evaluation of Epidemiologic Evidence	Low					
Expert Panel Evaluation of Toxicologic Evidence	Moderate					
Probability of Causation	20-39%					
Percentile of Exposure	0-9	10-24	25-49	50-74	75-89	>90
Percentile Assumed	0	10	25	50	75	90
Total DEHP metabolites, nmol/L	0	52.91	97.07	75.999	134.70	225.69
Unexposed Incidence	0.003					
Incremental Incidence	0	0.002	0.002	0.002	0.003	0.003
Attributable Cases	10,151					
Attributable Costs	$5.7 billion					

References

1. Trasande, L.; Zoeller, R.; Hass, U.; Kortenkamp, A.; Grandjean, P.; Myers, J.; DiGangi, J.; Bellanger, M.; Hauser, R.; Legler, J.; *et al.* Estimating Burden and Disease Costs of Exposure to Endocrine-Disrupting Chemicals in the European Union. *J. Clin. Endocrinol. Metab.* **2015**, *100*, 1245–1255, doi: 10.1210/jc.2014-4324.
2. Haines, D. A.; Saravanabhavan, G.; Werry, K. and Khoury, C. An overview of human biomonitoring of environmental chemicals in the Canadian Health Measures Survey: 2007–2019. *Int. J. Hyg. Environ. Health* **2017**, *220*, 13–28, doi: 10.1016/j.ijheh.2016.08.002.
3. Chen, Aimin; Yolton, K.; Rauch, S.; Webster, G.; Hornung, R.; Sjödin, A.; Dietrich, K.; Lanphear, B. Prenatal Polybrominated Diphenyl Ether Exposures and Neurodevelopment in U.S. Children through 5 Years of Age: The HOME Study. *Environ. Health Perspect* **2014**, 122, 856–862, doi: 10.1289/ehp.1307562.
4. Statistics Canada. Census Program. January 2001. https://www12.statcan.gc.ca/census-recensement/index-eng.cfm?MM=1 (accessed on 29 March, 2020).
5. Elise G. Childhood lead poisoning: conservative estimates of the social and economic benefits of lead hazard control. *Environ. Health Perspect* **2009**, *117*, 1162–1167, doi: 10.1289/ehp.0800408.
6. Honeycutt, A.; Dunlap, L.; Chen, H.; Homsi, G.; Grosse, S.; Schendel, D. Economic Costs of Mental Retardation, Cerebral Palsy, Hearing Loss, and Vision Impairment - United States, 2003. *Res Soc Sci Disabil* **2004**, *53*, 57–9.
7. OECD. OECD Data - Finance. https://data.oecd.org/searchresults/?hf=20&b=0&r=%2Bf%2Ftype%2Findicators&r=%2Bf%2Ftopics_en%2Ffinance&l=en&s=score (accessed 29 March, 2020).
8. Bellinger, D.; Croft C. A strategy for comparing the contributions of environmental chemicals and other risk factors to neurodevelopment of children. *Environ. Health Perspect.* **2012**, *120*, 501–507, doi: 10.1289/ehp.1104170.
9. Engel, S.; Wetmur, J.; Chen, J.; Zhu, C.; Barr, D.; Canfield, R.; Wolff, M. Prenatal exposure to organophosphates, paraoxonase 1, and cognitive development in childhood," *Environ. Health Perspect.* **2011**, *119*, 1182–1188, doi: 10.1289/ehp.1003183.
10. Bouchard, M.; Chevrier, J.; Harley, K.; Kogut, K.; Vedar, M.; Calderon, N.; Trujillo, C.; Johnson, C.; Bradman, A.; Barr, D.; Eskenazi, B. Prenatal exposure to organophosphate pesticides and IQ in 7-year-old children," *Environ. Health Perspect.*, **2011**, *119*, 1189–1195, doi: 10.1289/ehp.1003185.
11. United States Department of Labor, Bureau of Labor Statistics. Consumer Price Index. http://www.bls.gov/cpi/ (accessed 01 February, 2020).
12. Miodovnik, A.; Engel S.; Zhu C.; Ye, X.; Soorya, L.; Silva, M.; Calafat, A.; Wolff, M. Endocrine disruptors and childhood social impairment. *Neurotoxicology* **2011**, *32*, 261–267, doi: 10.1016/j.neuro.2010.12.009.
13. Ofner, M.; Coles, A. and Decou, M. L. *Autism Spectrum Disorder Among Children and Youth in Canada 2018: a Report of the National Autism Spectrum Disorder Surveillance System*. 2018. Accessed: 03 July, 2020. [Online]. Available: https://www.deslibris.ca/ID/10096072
14. Buescher, A.; Cidav, Z.; Knapp, M.; Mandell, D. Costs of autism spectrum disorders in the United Kingdom and the United States. *JAMA Pediatr.*, **2014**, *168*, 721–728, doi: 10.1001/jamapediatrics.2014.210.
15. Levin, M. The occurrence of lung cancer in man. *Acta - Unio Int. Contra Cancrum*, **1953**, *9*, 531–541.
16. Gascon, M.; Vrijheid, M.; Martínez, D.; Forns, J.; Grimalt, J.; Torrent, M.; Sunyer, J. Effects of pre and postnatal exposure to low levels of polybromodiphenyl ethers on neurodevelopment and thyroid hormone levels at 4 years of age. *Environ. Int.* **2011**, *37*, 605–611, doi: 10.1016/j.envint.2010.12.005.
17. Vasiliadis, H.-M.; Diallo, F.B.; Rochette L.; Smith M.; Langille, D.; Lin E.; Kisely, S.; Fombonne, E.;Thompson, A.H.; Renaud, J.; *et al.* Temporal Trends in the Prevalence and Incidence of Diagnosed ADHD in Children and Young Adults between 1999 and 2012 in Canada: A Data Linkage Study," *Can. J. Psychiatry Rev. Can. Psychiatr.* **2017**, *62*, 818–826, doi: 10.1177/0706743717714468.

18. Sikora, D. M.; Vora, P.; Coury, D. L. and Rosenberg, D. Attention-Deficit/Hyperactivity Disorder Symptoms, Adaptive Functioning, and Quality of Life in Children With Autism Spectrum Disorder. *Pediatrics* **2012**, *130*, S91–S97, doi: 10.1542/peds.2012-0900G.
19. Pelham, W. E. ; Foster, E. M. and Robb, J. A. The Economic Impact of Attention-Deficit/Hyperactivity Disorder in Children and Adolescents. *J. Pediatr. Psychol.* **2007**, *32*, 711–727, doi: 10.1093/jpepsy/jsm022.
20. Wu, H.; Bertrand, K.A.; Choi, A.L.; Hu, F.B.; Laden, F.; Grandjean, P.; Sun, Q. Persistent organic pollutants and type 2 diabetes: a prospective analysis in the nurses' health study and meta-analysis. *Environ. Health Perspect.* **2013**, *121*, 153–161, doi: 10.1289/ehp.1205248.
21. Turyk, M.; Anderson, H.; Knobeloch, L.; Imm, P.; Persky, V. Organochlorine exposure and incidence of diabetes in a cohort of Great Lakes sport fish consumers. *Environ. Health Perspect.* **2009**, *117*, 1076–1082, doi: 10.1289/ehp.0800281.
22. Public Health Agency of Canada. *Diabetes in Canada: facts and figures from a public health perspective.* Ottawa, Ont.: Public Health Agency of Canada, 2012. Accessed: 03 April, 2020. [Online]. Available: http://ra.ocls.ca/ra/login.aspx?inst=centennial&url=https://www.deslibris.ca/ID/232351
23. Rosella, L. C.; Lebenbaum, M.; Fitzpatrick, T.; O'Reilly, D.; Wang, J.; Booth, G. L.; Stukel, T. A.; Wodchis, W. P. Impact of diabetes on healthcare costs in a population-based cohort: a cost analysis. *Diabet. Med.* **2016**, *33*, 395–403, doi: 10.1111/dme.12858.
24. Statistics Canada. The Consumer Price Index. https://www150.statcan.gc.ca/n1/en/catalogue/62-001-X (accessed 31 March, 2020).
25. Iszatt, N.; Stigum, H.; Verner, M.-A.; White, R. A.; Govarts, E.; Murinova, L. P. ; Schoeters, G.; Trnovec, T.; Legler, J.;Pelé, F. *et al.* Prenatal and Postnatal Exposure to Persistent Organic Pollutants and Infant Growth: A Pooled Analysis of Seven European Birth Cohorts. *Environ. Health Perspect.*, **2015**, *123*, 730–736, doi: 10.1289/ehp.1308005.
26. Monteiro, P. O. A. and Victora, C. G. Rapid growth in infancy and childhood and obesity in later life--a systematic review. *Obes. Rev. Off. J. Int. Assoc. Study Obes.* **2005**, *6*, 143–154, doi: 10.1111/j.1467-789X.2005.00183.x.
27. Ong, K. K. and Loos, R. J. F. Rapid infancy weight gain and subsequent obesity: systematic reviews and hopeful suggestions. *Acta Paediatr. Oslo Nor. 1992*. **2006**, *95*, 904–908, doi: 10.1080/08035250600719754.
28. Rao, D. P.; Kropac, E.; Do, M. T.; Roberts, K. C. and Jayaraman, G. C. Childhood overweight and obesity trends in Canada. *Health Promot. Chronic Dis. Prev. Can. Res. Policy Pract.* **2016**, *36*, 194–198.
29. Valvi, D.; Mendez, M.; Martinez, D.; Grimalt, J.O.; Torrent, M.; Sunyer, J.; Vrijheid, M. Prenatal concentrations of polychlorinated biphenyls, DDE, and DDT and overweight in children: a prospective birth cohort study. *Environ. Health Perspect.* **2012**, *120*, 451–457, doi: 10.1289/ehp.1103862.
30. Finkelstein, E. A.; Graham, W. C. K. and Malhotra, R. Lifetime Direct Medical Costs of Childhood Obesity. *Pediatrics*, **2014**, *133*, 854–862, doi: 10.1542/peds.2014-0063.
31. Trasande, L.; Liu, Y.; Fryer, G. and Weitzman, M. Effects of childhood obesity on hospital care and costs, 1999-2005. *Health Aff. Proj. Hope*. **2009**, *28*, 751-760, doi: 10.1377/hlthaff.28.4.w751.
32. Trasande, L. and Chatterjee, S. The impact of obesity on health service utilization and costs in childhood. *Obes. Silver Spring Md*, **2009**, *17*, 1749–1754, doi: 10.1038/oby.2009.67.
33. Trasande, L. How Much Should We Invest In Preventing Childhood Obesity? *Health Aff. (Millwood)*, **2010**, *29*, 372–378.
34. Trasande, L. and Elbel, B. The economic burden placed on healthcare systems by childhood obesity. *Expert Rev. Pharmacoecon. Outcomes Res.*, **2012**, *12*, 39–45, doi: 10.1586/erp.11.93.
35. Song, Y.; Hauser, R.; Hu, F. B.; Franke, A. A.; Liu, S. and Sun, Q. Urinary concentrations of bisphenol A and phthalate metabolites and weight change: a prospective investigation in US women. *Int. J. Obes.* **2014**, *38*, 1532–1537, doi: 10.1038/ijo.2014.63.
36. Morin, S.; Tsang, J. F. and Leslie, W. D. Weight and body mass index predict bone mineral density and fractures in women aged 40 to 59 years. *Osteoporos. Int. J. Establ. Result Coop. Eur. Found. Osteoporos. Natl. Osteoporos. Found. USA*, **2009**, *20*, 363–370, doi: 10.1007/s00198-008-0688-x.
37. Statistics Canada. Mean height, weight, body mass index (BMI) and prevalence of obesity, by collection method and sex, household population aged 18 to 79, Canada, 2008, 2007 to 2009, and 2005," Jul. 2015. https://www150.statcan.gc.ca/n1/pub/82-003-x/2011003/article/11533/tbl/tbl1-eng.htm (accessed 02 April., 2020).
38. Janssen, I.; Lam, M. and Katzmarzyk, P. T. Influence of overweight and obesity on physician costs in adolescents and adults in Ontario, Canada. *Obes. Rev. Off. J. Int. Assoc. Study Obes.* **2009**, *10*, 51–57, doi: 10.1111/j.1467-789X.2008.00514.x.
39. Muennig, P.; Lubetkin, E.; Jia, H. and Franks, P. Gender and the Burden of Disease Attributable to Obesity. *Am. J. Public Health*, **2006**, *96*, 1662–1668, doi: 10.2105/AJPH.2005.068874.
40. Sun, Q.; Cornelis, M.C; Townsend, M.K; Tobias, D.; Eliassen, A. H.; Franke, A.A; Hauser, R.; Hu, F.B Association of Urinary Concentrations of Bisphenol A and Phthalate Metabolites with Risk of Type 2 Diabetes: A Prospective Investigation in the Nurses' Health Study (NHS) and NHSII Cohorts. *Environ. Health Perspect.* **2014**, *122*, 616–623, doi: 10.1289/ehp.1307201.
41. Valvi, D.; Casas, M.; Mendez, M.A.; Ballesteros-Gmez, A.; Luque, N.; Rubio, S.; Sunyer, J.; Vrijheid, M. Prenatal bisphenol a urine concentrations and early rapid growth and overweight risk in the offspring. *Epidemiol. Camb. Mass*. **2013**, *24*, 791–799, doi: 10.1097/EDE.0b013e3182a67822.
42. Ma, S. and Frick, K. D. A simulation of affordability and effectiveness of childhood obesity interventions. *Acad. Pediatr.* **2011**, *11*, 342–350, doi: 10.1016/j.acap.2011.04.005.
43. Vafeiadi, M.; Roumeliotaki, T.; Myridakis, A.; Chalkiadaki, G.; Fthenou, E.; Dermitzaki, E.; Karachaliou, M.; Sarri, K.; Vassilaki, M.; Stephanou, E. *et al.* Association of early life exposure to bisphenol A with obesity and cardiometabolic traits in childhood," *Environ. Res.* **2016**, *146*, 379–387, doi: 10.1016/j.envres.2016.01.017.

44. Kuhle, S.; Kirk, S.; Ohinmaa, A.; Yasui, Y.; Allen, A. C. and Veugelers, P. J. Use and cost of health services among overweight and obese Canadian children. *Int. J. Pediatr. Obes.* **2011**, *6*, 142–148, doi: 10.3109/17477166.2010.486834.
45. Tarride J.-E.; Haq, M.; Taylor, V.H.; Sharma, A.M.; Nakhai-Pour, H.R.; O'Reilly, D.; Xie, F.; Dolovich, L.; Goeree, R. Health status, hospitalizations, day procedures, and physician costs associated with body mass index (BMI) levels in Ontario, Canada. *Clin. Outcomes Res. CEOR*, **2012**, *4*, 21–30, doi: 10.2147/CEOR.S24192.
46. Hardell, L.; Bavel, B.; Lindström, G.; Eriksson, M. and Carlberg, M. In utero exposure to persistent organic pollutants in relation to testicular cancer risk. *Int. J. Androl.* **2006**, *29*, 228–234, doi: 10.1111/j.1365-2605.2005.00622.x.
47. Canadian Cancer Society, "Canadian Cancer Society's Steering Committee: Canadian Cancer Statistics 2010.," Canadian Cancer Society, Toronto, 2010. [Online]. Available: https://www.cancer.ca/~/media/cancer.ca/CW/cancer%20information/cancer%20101/Canadian%20cancer%20statistics/Canadian-Cancer-Statistics-2010-EN.pdf?la=en
48. de Oliveira, C. Phase-specific and lifetime costs of cancer care in Ontario, Canada. *BMC Cancer*, **2016**, 16, doi: 10.1186/s12885-016-2835-7.
49. Kiviranta, H.; Main, K.M.; Vartiainen, T.; Skakkebaek, N.E.; Virtanen, H.E.;Toppari, J.; Tuomisto, J.T.; Sundqvist, E.;Tuomisto, J. Flame retardants in placenta and breast milk and cryptorchidism in newborn boys. *Environ. Health Perspect.* **2007**, *115*, 1519–1526, doi: 10.1289/ehp.9924.
50. Mannetje, A. 't; Coakley, J.; Mueller, J. F.; Harden, F.; Toms, L.-M. and Douwes, J. Partitioning of persistent organic pollutants (POPs) between human serum and breast milk: a literature review. *Chemosphere* **2012**, *89*, 911–918, doi: 10.1016/j.chemosphere.2012.06.049.
51. Hsieh, M. H.; Roth, D. R. and Meng, M. V. Economic analysis of infant vs postpubertal orchiopexy to prevent testicular cancer. *Urology*, **2009**, *73*, 776–781, Apr. doi: 10.1016/j.urology.2008.10.059.
52. Meeker, J. D. and Ferguson, K. K. Urinary Phthalate Metabolites Are Associated With Decreased Serum Testosterone in Men, Women, and Children From NHANES 2011–2012. *J. Clin. Endocrinol. Metab.* **2014**, *99*, 4346–4352, doi: 10.1210/jc.2014-2555.
53. Araujo, A. B.; Dixon, J. M.; Suarez, E. A.; Murad, M. H.; Guey, L. T. and Wittert, G. A. Clinical review: Endogenous testosterone and mortality in men: a systematic review and meta-analysis. *J. Clin. Endocrinol. Metab.*, **2011**, *96*, 3007–3019, doi: 10.1210/jc.2011-1137.
54. Statistics Canada, "Trends in mortality rates, 2000 to 2013," Feb. 07, 2017. https://www150.statcan.gc.ca/n1/pub/82-625-x/2017001/article/14775-eng.htm (accessed 20 September, 2020).
55. Max, W. Present Value of Lifetime Earnings, 2009. Unpublished Tables. Institute for Aging, University of California, San Francisco, 2013.
56. Buck Louis, G. M.; Sundaram, R. ; Sweeney, A. M.; Schisterman, E. F.; Maisog, J. and Kannan, K. Urinary bisphenol A, phthalates, and couple fecundity: the Longitudinal Investigation of Fertility and the Environment (LIFE) Study. *Fertil. Steril.* **2014**, *101*, 1359–1366, doi: 10.1016/j.fertnstert.2014.01.022.
57. Gunby, J. Assisted reproductive technologies (ART) in Canada: 2010 results from the Canadian ART Register. IVF Directors Group of the Canadian Fertility and Andrology Society, Montreal, Quebec, Canada. Accessed: 25 September, 2020. [Online]. Available: https://cfas.ca/_Library/_documents/CARTR_2010.pdf
58. Bushnik, T.; Cook, J. L.; Yuzpe, A. A.; Tough, S. and Collins, J. Estimating the prevalence of infertility in Canada. *Hum. Reprod. Oxf. Engl.* **2012**, *27*, 738–746, doi: 10.1093/humrep/der465.
59. Zhang, J. and Yu, K. F. What's the Relative Risk?A Method of Correcting the Odds Ratio in Cohort Studies of Common Outcomes. *JAMA*, **1998**, *280*, 1690–1691, doi: 10.1001/jama.280.19.1690.
60. United Nations Population Division, "World Contraceptive Use. 2014. http://data.un.org/DocumentData.aspx?id=356 (accessed 25 September 2020).
61. Statistics Canada, Marital Status (13), Age (16) and Sex (3) for the Population 15 Years and Over of Canada, Provinces and Territories and Census Metropolitan Areas, 1996 to 2016 Censuses - 100% Data. 2019. https://www12.statcan.gc.ca/census-recensement/2016/dp-pd/dt-td/Rp-eng.cfm?LANG=E&APATH=3&DETAIL=0&DIM=0&FL=A&FREE=0&GC=0&GID=0&GK=0&GRP=1&PID=109650&PRID=10&PTYPE=109445&S=0&SHOWALL=0&SUB=0&Temporal=2016&THEME=117&VID=0&VNAMEE=&VNAMEF= (accessed 20 September, 2020).
62. Chambers, G. M.; Sullivan, E. A.; Ishihara, O.; Chapman, M. G. and Adamson, G. D. The economic impact of assisted reproductive technology: a review of selected developed countries. *Fertil. Steril.* **2009**, *91*, 2281–2294, doi: 10.1016/j.fertnstert.2009.04.029.
63. Trabert, B.; Chen, Z.; Kannan, K.;Peterson, C.M.;Pollack, A.Z.; Sun, L.; Buck Louis, G.M. Persistent organic pollutants (POPs) and fibroids: results from the ENDO study. *J. Expo. Sci. Environ. Epidemiol.* **2015**, *25*, 278–285, doi: 10.1038/jes.2014.31.
64. Marshall, L.M.; Spiegelman, D.; Barbieri, R.L.; Goldman, M.B.; Manson, J.E.; Colditz, G.A.; Willett, W.C.; Hunter, D.J. Variation in the incidence of uterine leiomyoma among premenopausal women by age and race. *Obstet. Gynecol.* **1997**, *90*, 967–973, doi: 10.1016/s0029-7844(97)00534-6.
65. Zimmermann, A.; Bernuit, D.; Gerlinger, C.; Schaefers, M. and Geppert, K. Prevalence, symptoms and management of uterine fibroids: an international internet-based survey of 21,746 women. *BMC Womens Health*, **2012**, *12*, 6, doi: 10.1186/1472-6874-12-6.

66. Soliman, A. M.; Yang, H.; Du, E. X.; Kelkar, S. S. and Winkel, C. The direct and indirect costs of uterine fibroid tumors: a systematic review of the literature between 2000 and 2013. *Am. J. Obstet. Gynecol.*, **2015**, *213*, 141–160, doi: 10.1016/j.ajog.2015.03.019.
67. Al-Fozan, H.; Dufort, J.; Kaplow, M.; Valenti, D. and Tulandi, T. Cost analysis of myomectomy, hysterectomy, and uterine artery embolization," *Am. J. Obstet. Gynecol.* **2002**, *187*, 1401–1404, doi: 10.1067/mob.2002.127374.
68. Louis, G.M.B; Peterson, C.M.; Chen, Z.; Croughan, M.; Sundaram, R.; Stanford, J.; Varner, M.W.; Kennedy, A.; Giudice, L.; Fujimoto, V.Y. *et al.* Bisphenol A and phthalates and endometriosis: the Endometriosis: Natural History, Diagnosis and Outcomes Study. *Fertil. Steril.* **2013**, *100*, 162-169. doi: 10.1016/j.fertnstert.2013.03.026.
69. Missmer, S. A.; Hankinson, S. E.; Spiegelman, D.; Barbieri, R. L.; Marshall, L. M. and Hunter, D. J. Incidence of laparoscopically confirmed endometriosis by demographic, anthropometric, and lifestyle factors. *Am. J. Epidemiol.* **2004**, *160*, 784–796, doi: 10.1093/aje/kwh275.
70. Levy, A.R.; Osenenko, K.M.; Lozano-Ortega, G.; Sambrook, R.; Jeddi, M.;Bélisle, S.; Reid, R.L. Economic burden of surgically confirmed endometriosis in Canada. *J. Obstet. Gynaecol. Can. JOGC J. Obstet. Gynecol. Can. JOGC*, **2011**, *33*, 830–837, doi: 10.1016/S1701-2163(16)34986-6.
71. Murray, C.J.L.;Vos, T.; Lozano, R.; AlMazroa, M.A.; Memish, Z.A. Disability-adjusted life years (DALYs) for 291 diseases and injuries in 21 regions, 1990-2010: a systematic analysis for the Global Burden of Disease Study 2010. *Lancet Lond. Engl.*, **2012**, *380*, 2197–2223, doi: 10.1016/S0140-6736(12)61689-4.
72. Constantino, J. and Gruber, C. Social Responsiveness Scale (SRS). Western Psychological Services, 2005.
